# Supplementary material for: Simple scaling laws control the genetic architectures of human complex traits
Source: PLoS Biol. 2025 Oct 13;23(10):e3003402. doi: 10.1371/journal.pbio.3003402 (PMC12517483; doi:10.1371/journal.pbio.3003402)
Supplement: S1 Note — Fig A. Geometric relationship between the n-dimensional effect and its projection on a single trait. Fig B. Mutation-selection balance approximation. Fig C. Histogram of d values for all GWAS hits used. Fig D. MAF and z-score bound the selection coefficient. Fig E. Demographic model. Fig F. CDFs of the inferred distribution of selection coefficients for all 95 traits. Fig G. Tuning the strength of the penalty. Fig H. Choosing the number of knots. Fig I. Lowered sample sizes. Fig J. Heritability estimates. Fig K. Proportion of rare variants. Fig L. Inference on simulated datasets. Fig M. Inference on a non-SSD dataset. Fig N. SSD results for 15 nearly independent traits. Fig O. Summaries of GWAS hits for the 95 traits. Fig P. Relative Uncertainty. Fig Q. Maximal correlation. Fig R. Effect of assortative mating. Fig S. The genetic architecture and inference for simulated architectures. Fig T. Simulation with 9,834 hits. Fig U. Schematic of mutation origin on terminal branch. Fig V. Distribution of allele ages. Fig W. Heuristic estimators of allele ages. Fig X. Prediction of allele ages with shifted distributions of selection coefficients. Table A. Outlier SNPs. Table B. Model and inference assumptions. (PDF) [file pbio.3003402.s001.pdf]

Simple scaling laws for the genetic architectures of human complex traits
Supplementary note

Yuval B. Simons[*,1,2,3], Hakhamanesh Mostafavi[3,4,5], Huisheng Zhu[6], Courtney J. Smith[3], Jonathan K. Pritchard[*,3,6], and Guy Sella[*,7,8]

[1] Section of Genetic Medicine, University of Chicago, Chicago, Illinois, United States of America.
[2] Department of Human Genetics, University of Chicago, Chicago, Illinois, United States of America.
[3] Department of Genetics, Stanford University, Stanford, California, United States of America.
[4] Center for Human Genetics and Genomics, New York University School of Medicine, New York City, New York, United States of America.
[5] Department of Population Health, New York University School of Medicine, New York City, New York, United States of America.
[6] Department of Biology, Stanford University, Stanford, California, United States of America.
[7] Department of Biological Sciences, Columbia University, New York City, New York, United States of America.
[8] Program for Mathematical Genomics, Columbia University, New York City, New York, United States of America.
[*] yuval.simons@uchicago.edu (YBS), pritch@stanford.edu (JKP), gs2747@columbia.edu (GS)

September 18, 2025

Contents

1 The Model **4**
1.1 Selection on Traits . . . 4
1.2 Selection on Variants . . . 5
1.3 Relationship between selection coefficients and effect sizes . . . 7
1.4 Genome-wide architecture . . . 8
1.5 Reparameterization in terms of h^2/L . . . 9
1.6 Summary of model . . . 11

2 The Likelihood **11**
2.1 Minor allele frequencies . . . 11
2.2 The estimated effect size . . . 11

2.3 z-score 12
2.4 Varying imputation quality 12
2.5 The joint distribution of MAFs and z-scores 13
2.6 Threshold for genome-wide significance 14
2.7 The conditional co-distribution of MAFs and z-scores 14
2.8 Estimating model parameters 14
2.9 Effects of joint effects size estimation on the standard error 15
2.10 How do MAFs and z-scores translate to selection coefficients? 16

3 Maximizing the likelihood **17**

3.1 Binning and gridding 18
3.2 Site frequency spectra 18
3.3 Precaculated probabilities 19
3.4 Calculating the likelihood via linear algebra 19
3.5 Estimating C for a given $f(s)$ 20
3.6 Estimating $f(s)$ from the marginal likelihood 20
3.7 Maximizing the likelihood for a single shared distribution of s 21
3.8 Adding a penalty to regularize $f(s)$ 21
3.9 Estimating confidence intervals 22

4 Estimating model fit **22**

4.1 The residual p-value 22
4.2 Cross-validated residual p-value 23
4.3 Measure of model fit for a trait 23
4.4 Measure of model fit for variants 23
4.5 Accounting for outlier variants during inference 24
4.6 Residual p-values for alternative models 24
4.7 Tuning ϵ 25
4.8 Choosing the number of knots 25
4.9 Consistency with lower sample sizes 26
4.10 Consistency with twin and SNP heritabilities 26
4.11 Contribution of rare variants 27
4.12 TSD vs. SSD fit for a trait 28

5 Validating our inference using simulations **28**

5.1 Simulating a single trait 29

5.2 Simulating sets of traits 29
5.3 Validating our inference on simulated traits 29

6 UKBB Dataset **31**
6.1 GWAS summary statistics for diverse traits 31
6.2 Trait choice 31
6.3 Variant filtering 32
6.4 Subset of independent traits 32
6.5 Summaries of GWAS hits 33
6.6 Functional followup of outlier variants 34
6.7 Computational reduction in study size 34

7 Are GWAS hits a good proxy for the underlying causal variants? **35**
7.1 How does the choice of tagging variants affect our estimates of frequency and effect size? 35
7.1.1 Fine-mapped traits in the UK biobank 35
7.1.2 The reason choice of tagging variants is a small effect 36
7.2 How well does COJO capture the number, frequencies, and effects of hits? 37
7.2.1 Simulating hight-like genetic architectures 37
7.2.2 Simulation results and the effect of assortative mating 38
7.2.3 Simulation without assortative mating 38
7.2.4 Tagging of causal variants 39
7.2.5 Inference on simulation 39
7.3 Are our estimates of the number, frequencies, and effects of hits biased by linked small effect variants? 40
7.3.1 Simulating hight-like genetic architectures using the GIANT dataset 41
7.3.2 Expected architecture 41
7.3.3 Results 41

8 Allele ages **42**
8.1 RELATE output 42
8.2 GWAS hits are younger than matched controls 43
8.3 Allele age prediction 44
8.4 Bias in allele age estimation 44
8.5 Heuristic point estimate of allele age 45
8.6 Sensitivity to estimates of s 46

9 Parameter values in main text figures **46**
9.1 Parameter values for Figure 1 46
9.2 Parameter values for Figure 2 46
9.3 Parameter values for Figure 6 47

10 Similarities in genetic architectures after scaling **47**

11 Supplementary tables **55**
11.1 Table A: Outlier SNPs 55
11.2 Table B: Model and inference assumptions 56

1 The Model

We will present here a description of the pleiotropic stabilizing selection model used in the paper. It is an extension, and to a certain degree reparameterization, of the model presented in Simons et al 2018 [1]. Like the 2018 model, we focus on one focal trait under stabilizing selection and assume that variants may have pleiotropic effects on other, non-measured traits that also experience stabilizing selection.

We describe how the selection on a site depends on its effect on all traits. This allows us to arrive at expressions for the distribution of allele frequencies and effect sizes at trait-affecting sites, conditional on their selection coefficients. We then consider the existence of L such sites, with L being the mutational target size for the trait in question, and we denote the distribution of selection coefficient at such sites as $f(s)$. We show that, if effect sizes are measured in units of the phenotypic standard deviation, the last parameter needed to fully characterize the architecture is the heritability over the target size, i.e. the mean contribution to heritability per site.

Thus, our model describes the number of segregating sites affecting the trait, their frequencies and their effect sizes using L and $f(s)$ and h^2/L - the number of trait-affecting sites, the distribution of their selection coefficients and each site's mean contribution to heritability.

1.1 Selection on Traits

We want to look at traits under stabilizing selection, that is when extreme trait values have lowered fitness compared to some medium, optimal trait value. We denote the trait value as Y and we parametrize fitness as

$$W(Y) = \exp\left(-Y^2/2V_S\right)$$

where V_S is the width of the fitness function around the trait optimum, which we denote as the value $Y = 0$. Since under stabilizing selection models, phenotypes are tightly distributed around the optimum then this functional form can approximate any smooth and symmetric fitness function around the optimum.

The phenotype itself is modeled as an additive sum of genetic and environmental effects. The phenotype of an individual is a sum over the genetic contribution of genetic variants at many

trait-affecting sites plus an environmental effect:

$$Y = \sum_i b_i \cdot g_i + e$$

where b_i is the effect size of the derived allele at site i, $g_i = 0, 1, 2$ is the genoype at site i and $e \sim N(0, V_E)$ is the environmental effect.

There may be, and most probably is, more than one trait under stabilizing selection. We assume that stabilizing selection acts on n such traits, which we denote as Y_1,Y_2...Y_n, and that the fitness is multiplicative, i.e.

$$W(Y_1, Y_2...Y_n) = \exp(-Y_1^2/2V_S) \cdot \exp(-Y_2^2/2V_S) \cdots \exp(-Y_n^2/2V_S).$$

This equation can take a more succinct form if we think of the phenotype as a point in an n-dimensional trait space, i.e. an individual's phenotype is represented as the vector $\vec{Y} = (Y_1, Y_2, \ldots Y_n)$. We can now write

$$W(\vec{Y}) = \exp\left(-\|\vec{Y}\|^2/2V_S\right)$$

with $\vec{Y} = (Y_1, Y_2, \ldots Y_n)$ being a representation of the phenotype (i.e., the phenotypic value at all n traits) as a vector in an n-dimensional Euclidean trait space. As we discuss in Simons 2018, the fitness function around a peak can always be brought to this form using a linear transformation of the traits. We assume all variants affect the same number of traits and that all traits are identical, see Simons 2018 for discussion of the robustness of our results to violations of these assumptions.

Phenotypes can now be modeled as a vector additive sum of genetic and environmental effects. The phenotype of an individual is a sum over the genetic contribution of genetic variants at many trait-affecting sites plus an environmental effect:

$$\vec{Y} = \sum_i \vec{b_i} \cdot g_i + \vec{e}$$

where $\vec{b_i}$ is the effect size (row vector in in trait space) of the derived allele at site i – meaning that the projection of $\vec{b_i}$ on any given dimension is the effect size of the derived allele at site i on the trait represented by that dimension. $g_i = 0, 1, 2$ is still the genotype at site i and $\vec{e} \sim N(0, V_E \cdot I)$, with I being the n-dimensional identity matrix.

Since traits are sums of the small additive contributions of many alleles they will be approximately normally distributed in the population. As discussed in Simons 2018, we can assume that trait mean is at the optimum, which is denoted as $\vec{0}$, since stabilizing selection keeps it is very close to the optimum. We can therefore denote $\vec{Y} \sim N(0, V_P \cdot I)$.

1.2 Selection on Variants

How does selection on traits affect variant allele frequencies?

In order to answer this question, we will calculate the expected change in allele frequency per generation caused by stabilizing selection on traits. We do this by averaging over the possible genetic background for the three possible genotypes at a site. Let's look at single site. We can now separate the phenotype into three contributions: site i, the genetic background (all other sites), and the environmental contribution:

$$\vec{Y} = \vec{b_i} \cdot g_i + \sum_{j \neq i} \vec{b_j} \cdot g_j + \vec{e} = \vec{b_i} \cdot g_i + \vec{Y}_{\text{not i}}$$

where $\vec{Y}_{\text{not i}}$ captures all effects other than site i.

What is the distribution of $\vec{Y}_{\text{not i}}$? The contribution from site i has mean $\vec{b}_i \cdot 2q_i$ and variance $\text{diag}(\vec{b}_i^T \vec{b}_i) \cdot 2q(1-q)$. Together, the contribution from site i and $\vec{Y}_{\text{not i}}$ have mean $\vec{0}$ and variance $V_P \cdot I$ and therefore we can approximate

$$\vec{Y}_{\text{not i}} \sim N(-\vec{b}_i \cdot 2q_i, V_P \cdot I)$$

where we assume the contribution to variance from site i, $\text{diag}(\vec{b}_i^T \vec{b}_i) \cdot 2q(1-q)$, is small compared to V_P.

We can now calculate the mean fitness of each one of the three possible genotypes at site i. Let's look at individuals who are homozygous for the ancestral allele. Their phenotypes are just

$$\vec{Y}_{00} = \vec{Y}_{\text{not i}}$$

and so their mean fitness is

$$W_{00} = E\left[W(\vec{Y}_{\text{not i}})\right]$$

with $W(\vec{Y}) = \exp(-|\vec{Y}|^2/2V_S)$, as before, and the expectation taken over the distribution of $\vec{Y}_{\text{not i}}$. Similarly, for the heterozygote $\vec{Y}_{01} = \vec{Y}_{\text{not i}} + \vec{b}_i$ and therefore

$$W_{01} = E\left[W(\vec{Y}_{\text{not i}} + \vec{b}_i)\right]$$

and similarly

$$W_{11} = E\left[W(\vec{Y}_{\text{not i}} + 2\vec{b}_i)\right]$$

and the first moment of change in allele frequency is

$$E[\Delta q] = -pq\frac{p(W_{00} - W_{01}) + q(W_{01} - W_{11})}{\bar{W}}$$

with $\bar{W} = p^2 W_{00} + 2pqW_{01} + q^2 W_{11}$.

The expression for $E[\Delta q]$ greatly simplifies when $|\vec{b}_i|^2 \ll V_S$ and $V_P \ll V_S$, i.e. when the reduction in mean log fitness due to site i and the overall phenotypic variation is much smaller than 1. In this case the relative fitnesses are

$$\frac{W_{00}}{\bar{W}} \approx 1 - \frac{4q^2 \cdot |\vec{b}_i|^2}{2V_S}, \; \frac{W_{01}}{\bar{W}} \approx 1 - \frac{(p-q)^2 \cdot |\vec{b}_i|^2}{2V_S}, \; \frac{W_{11}}{\bar{W}} \approx 1 - \frac{4p^2 \cdot |\vec{b}_i|^2}{2V_S},$$

and

$$E[\Delta q] \approx -\frac{|\vec{b}_i|^2}{V_S} \cdot pq(\tfrac{1}{2} - q) = -s \cdot pq(\tfrac{1}{2} - q)$$

and we see that $s = \frac{|\vec{b}_i|^2}{V_S}$ serves as the selection coefficient acting on a variant.

Note that $E[\Delta q]$ takes the classic form for underdominant selection even though the relative fitnesses of the three genotypes do not. What we observe is an effective underdominant selection that comes about through the frequency dependency of the relative fitnesses.

The above equation for $E[\Delta q]$, together with demography, determines the distribution of derived allele frequencies for variants with a given selection coefficient, which we denote as $P(q|s)$. This distribution can be estimated via forward simulations (as we do) or solving the relevant Kolmogorov equation. [1]

[1] Although the Kolmogorov equation can usually only be solved numerically, for a constant population size an

1.3 Relationship between selection coefficients and effect sizes

Under our model, the strength of selection acting on a variant, i.e. its selection coefficient, is determined by the variant's phenotypic effect on all traits. However, for solving the model, it is convenient to express the distribution of effect size on a focal trait conditional on the selection coefficient, i.e. conditional on the overall effect on all traits. We obtain an expression for this distribution using purely geometric reasoning.

Variants with a given n-dimensional effect size correspond to a hypersphere in the n dimensional trait space with radius $|\vec{b}|$, see Fig. AA. Of those, variants with a given effect size on our focal trait, b_1, correspond to a cross-section of that sphere. The ratio of the cross-section's area to the sphere's area is the density of variants with effect size b_1.

We will now calculate this ratio for variants with effect size between b_1 and $b_1 + \Delta b_1$, with Δb_1 being arbitrarily small. The area of that sphere is proportional to its radius to the $(n-1)$th power, i.e. to $|\vec{b}|^{n-1}$. The area of the cross-section is proportional to its radius, which is $\sqrt{|\vec{b}|^2 - b_1^2}$, to the power of $n-2$ times $\Delta b_1 \cdot \frac{|\vec{b}|}{\sqrt{|\vec{b}|^2 - b_1^2}}$, the arc length corresponding to Δb_1, see Fig. AB.

Therefore, the ratio of the areas is

$$\frac{A_{\text{cross section}}}{A_{\text{sphere}}} \propto \frac{\left(\sqrt{|\vec{b}|^2 - b_1^2}\right)^{n-2} \cdot \Delta b_1 \cdot \frac{|\vec{b}|}{\sqrt{|\vec{b}|^2 - b_1^2}}}{|\vec{b}|^{n-1}} = \left(\sqrt{1 - \frac{b_1^2}{|\vec{b}|^2}}\right)^{\frac{n-3}{2}} \cdot \frac{\Delta b_1}{|\vec{b}|}$$

The density of b_1 conditional on $|\vec{b}|$ will then be

$$f(b_1) \propto \left(\sqrt{1 - \frac{b_1^2}{|\vec{b}|^2}}\right)^{\frac{n-3}{2}} \cdot \frac{1}{|\vec{b}|}.$$

This somewhat daunting expression greatly simplifies when $n \gg 1$. In this limit, we know that the squared effect on the focal trait is of the order of $|\vec{b}|^2/n$ making $b_1^2/|\vec{b}|^2 \sim O(1/n)$. In addition, since $n \gg 1$ then $(n-3)/2 \approx n/2$. Taking these simplifications together we get that

$$f(b_1) \propto \left(\sqrt{1 - \frac{b_1^2}{|\vec{b}|^2}}\right)^{\frac{n-3}{2}} \cdot \frac{1}{|\vec{b}|} \approx \left(\sqrt{1 - \frac{b_1^2}{|\vec{b}|^2}}\right)^{\frac{n}{2}} \cdot \frac{1}{|\vec{b}|} \approx \exp\left(-\frac{n}{2} \cdot \frac{b_1^2}{|\vec{b}|^2}\right) \cdot \frac{1}{|\vec{b}|} = \frac{1}{\sqrt{|\vec{b}|^2}} \cdot \exp\left(-\frac{1}{2} \cdot \frac{b_1^2}{|\vec{b}|^2/n}\right).$$

which we immediately recognize as a Gaussian function meaning that

$$b_1 \sim N\left(0, \frac{|\vec{b}|^2}{n}\right).$$

Since effect sizes on a focal trait are normally distributed conditional on the n-dimensional effect, they are also normally distributed conditional on the selection coefficient (since $s = |\vec{b}|^2/V_S$):

$$b_1 \sim N\left(0, \frac{V_S}{n} \cdot s\right).$$

analytical solution exists, see Simons 2018. For a large population size, it can be shown that this analytical solution for the minor allele frequency takes the simple form

$$P(x|s) = 2N_e\mu \cdot \frac{2\exp(-2N_e s \cdot x(1-x))}{x(1-x)}$$

for $x > 1/2N_e$, with x being the minor allele frequency. Though we don't use it in this work we thought it could be a useful result to mention.

Because this result relies on the geometry of high dimensional spaces it holds even when we relax the assumption of complete isotropy, albeit with an effective dimension replacing n in the above equation. See Simons (2018) for more details. Therefore, when looking at a given trait, we can drop the subscript of b_1 and arrive at

$$P(b|s) = \frac{1}{\sqrt{2\pi V_S/n}} \cdot \exp\left(-\frac{1}{2} \cdot \frac{b_1^2}{V_S/n}\right).$$

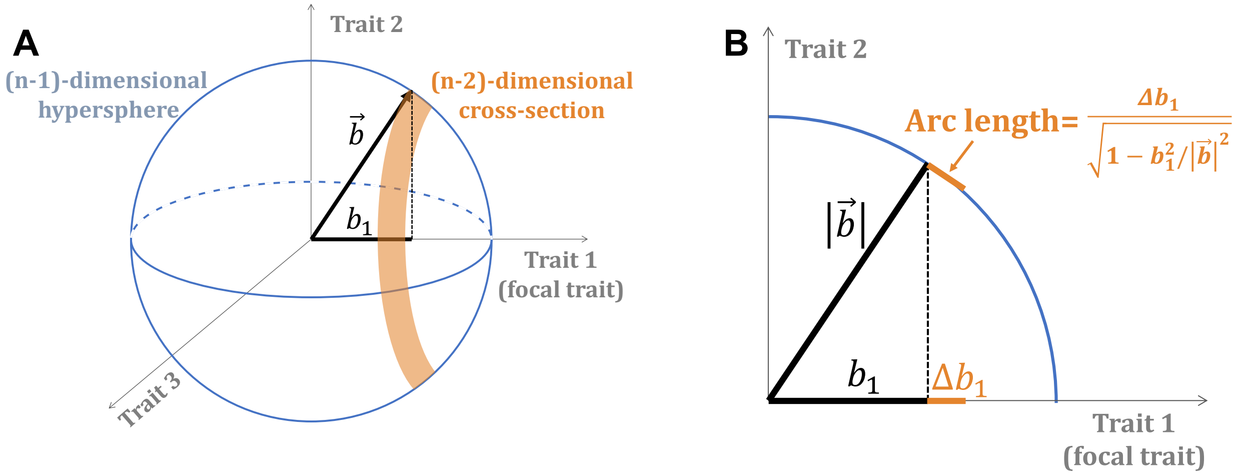

Figure A: **Geometric relationship between the n-dimensional effect and its projection on a single trait.** ***(A)*** *The possible values of $\vec{b}$ for a given $|\vec{b}|$ form an $n-1$-dimensional hypersphere in the n dimensional trait space. The values of $\vec{b}$ corresponding to a given effect size on the focal trait, b_1, form a cross-section of the hypersphere.* ***(B)*** *The geometry of calculating the arc length of the cross section.*

1.4 Genome-wide architecture

We can now combine the above results to calculate the distribution of derived allele frequency, q, and effect size, b, for L sites affecting the trait (i.e., L is the mutational target size for the trait). The density of sites with a given q & b for a given selection coefficient s is given by

$$P(q, b|s) = P(q|s) \cdot P(b|s)$$

with $P(q|s)$ and $P(b|s)$ as given above. Note that, conditional on the selection coefficient, the frequency and effect size are independent.

The overall distribution of q & b at all L sites is given averaging over the distribution of selection coefficients $f(s)$

$$P(q, b) = \int_s P(q, b|s) \cdot f(s) \cdot ds$$

Averages over the distribution of frequencies and effect sizes can then be calculated as

$$E\left[g(q, b)\right] = \int_q \int_b g(q, b) \cdot P(q, b) \cdot dbdq$$

with g being any function of q & b. Expectation of sums over all sites can be calculated by

$$E\left[\sum_i g(q_i, b_i)\right] = L \cdot E\left[g(q, b)\right].$$

To be concrete we will give a few useful examples:

- The probability of a variant being above a given frequency threshold q^* is
  $P(q > q^*) = E\left[\mathbb{1}_{q>q^*}\right] = \int_q \int_b \mathbb{1}_{q>q^*} \cdot P(q,b) \cdot dbdq = \int_{q>q^*} \int_b P(q,b) \cdot dbdq$
  and therefore the number of variants above that threshold is
  $\#_{q>q^*} = L \cdot P(q > q^*)$.
- The mean effect on the phenotype of variation at site is
  $E[2q \cdot b] = \int_q \int_b 2q \cdot b \cdot P(q,b) \cdot dbdq$
  and the overall mean genetic contribution to the phenotype is
  $E[Y] = L \cdot E[2q \cdot b]$.
  Without mutational bias or directional selection both of these expected values are equal to zero.
- The mean contribution to phenotypic variance from a site is
  $E[2q(1-q) \cdot b^2] = \int_q \int_b 2q(1-q) \cdot b^2 \cdot P(q,b) \cdot dbdq$
  and therefore the genetic contribution to variance is
  $V_G = L \cdot E[2q(1-q) \cdot b^2]$.

1.5 Reparameterization in terms of h^2/L

When looking at single sites, we have expressed the relation between selection and effect size in terms of the width of the fitness function around the optimum, V_S, and the degree of pleiotropy, i.e. number of traits n. Looking at all sites, we can express this relation using the heritability h^2 and the target size L, allowing us to better interpret our results.

Under our model, the contribution to genetic variance from all sites is

$$V_G = L \cdot E[2q(1-q) \cdot b^2]$$

and when measuring effect size in units of the phenotypic standard deviation this equation scales to

$$h^2 = V_G/V_P = L \cdot E[2q(1-q) \cdot \beta^2]$$

with

$$\beta = b/\sqrt{V_P}.$$

Therefore,

$$\frac{h^2}{L} = E[2q(1-q) \cdot \beta^2]$$

and we see that the heritability over the target size is the expected contribution to heritability from a single site, i.e. the heritability per site. Conditional on the selection coefficient, effect sizes and allele frequencies are independently distributed. We can take the expectation over the distribution of effect sizes conditional on the selection coefficient – since $E\left[\beta^2|s\right] = (V_S/n) \cdot s$ then $E\left[\beta^2|s\right] = (V_S/(n \cdot V_P)) \cdot s$. Therefore,

$$\frac{h^2}{L} = \frac{V_S}{n \cdot V_P} E[2q(1-q) \cdot s] = \frac{E[2q(1-q) \cdot s]}{n \cdot V_P/V_S}.$$

We have now arrived at a second interpretation of the heritability per site. For small selection coefficients, the numerator on the right hand side of the above equation is the mean reduction in mean log fitness due to a single site. The denominator is the reduction in mean log fitness due to the overall variation in phenotype (in all n traits). So the heritability per site is both the mean relative contribution of single site to genetic variance and the mean relative contribution of a single site to the reduction in log fitness.

Under mutation-selection balance $E[2q(1-q)\cdot s] = 4\mu$, with μ being the mutation rate. As you can see in Fig. BA, this relation approximately holds for single selection coefficients as long as selection is comparable with genetic drift under a European-like demographic history (see in depth discussion of the effects of demography in [2]). Therefore, the relationship $E[2q(1-q)\cdot s] \approx 4\mu$ holds in general (see BB), albeit with a small dependency on the distribution of selection coefficients.[2]

We can therefore write

$$\frac{V_S}{n \cdot V_P} = \frac{k}{4\mu} \cdot \frac{h^2}{L}$$

with

$$k \equiv \frac{4\mu}{E[2q(1-q)\cdot s]} \approx 1$$

and therefore

$$\beta \sim N\left(0, \frac{k}{4\mu} \cdot \frac{h^2}{L} \cdot s\right)$$

and we see that the heritability per site sets the scale of effect sizes in units of the phenotypic standard deviation.

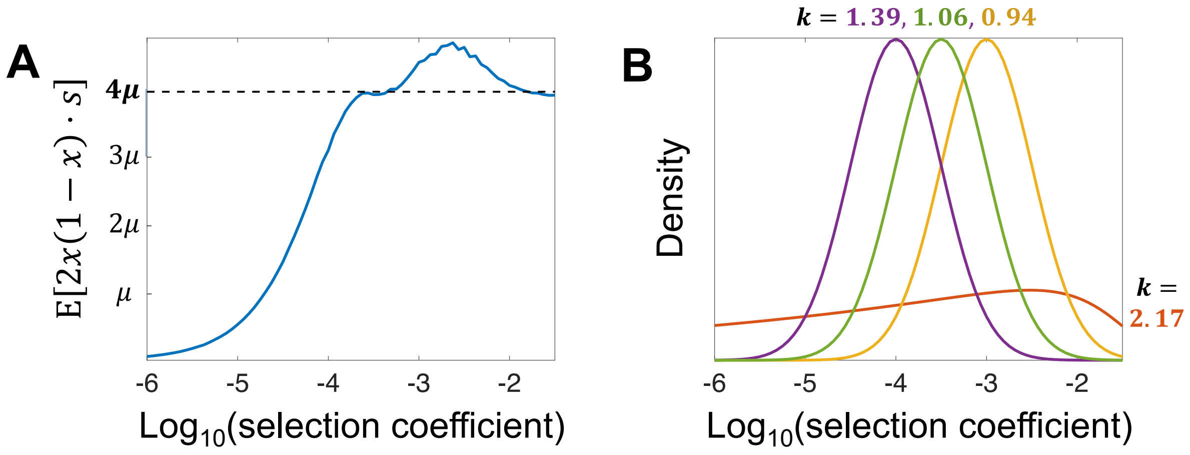

Figure B: **Mutation-selection balance approximation.** **(A)** *For a single selection coefficient, the approximation $E[2q(1-q)\cdot s] \approx 4\mu$ holds for $s \geq 10^{-4}$ even with non-equilibrium demography. The scale of $E[2q(1-q)\cdot s]$ is set by 4μ even for $s < 10^{-4}$.* ***(B)*** *As a result, we can define $E[2q(1-q)\cdot s] = 4\mu/k$ for a distribution of selection coefficients, and we see that for all distributions k is of the order of 1. Figure data available at: https://doi.org/10.5281/zenodo.17041176.*

[2]The only exception is if the bulk of the distribution comes from the effectively neutral region, in which case $E[2q(1-q)\cdot s] \ll 4\mu$. However, in this case allele frequencies are determined solely by genetic drift and therefore there is no relation between frequencies and effect sizes. This scenario is clearly contraindicated by data.

1.6 Summary of model

We have described a model with three inputs: the number of trait-affecting sites, or mutational target size, denoted as L; the distribution of selection coefficients at trait-affecting sites, denoted as $f(s)$; and the mean contribution to heritability of a trait-affecting site, denoted as h^2/L. New mutations arise at the L sites, thus sampling selection coefficients from $f(s)$.

The distribution of derived allele frequencies at a site is determined by the selection coefficient and the population's demography and we denote it as $P(q|s)$. The distribution of effect sizes (in units of the phenotypic standard deviation), which we denote as $P(\beta|s)$, is a normal distribution with mean zero and variance approximately equal to $1/4\mu \cdot h^2/L \cdot s$. The distribution of any summary of allele frequency and effect sizes follows from these distributions.

2 The Likelihood

We will present here an overview of our method to infer the three components of our model - L, $f(s)$ and h^2/L - from the co-distribution of minor allele frequencies and z-scores of GWAS hits. To do this, we first recast our model from derived allele frequencies and effect sizes to minor allele frequencies and z-scores, which allows us to condition on variants being GWAS hits. We then write a composite log-likelihood for L, $f(s)$ and h^2/L given the minor allele frequencies and z-scores of GWAS hits. Lastly, we tweak this likelihood to account for varying imputation qualities among SNPs.

2.1 Minor allele frequencies

In order to simplify our equations and avoid having to account for errors in identifying the derived allele frequency of the causal variant tagged by a GWAS hit, we fold the frequency spectrum, i.e. work exclusively with minor allele frequencies (MAFs) in our likelihood. The minor allele frequency is defined as

$$x = min(q, 1-q)$$

and its distribution is

$$P(x|s) = P(q|s) + P(1-q|s).$$

The MAF can take values between 0 and 0.5. Due to the decline in imputation quality at low MAFs, we will restrict ourselves to sites with $x > 0.01 = 1\%$. At frequencies above 1% and with study sizes above 100k, the estimation error for x is very small ($\Delta x/x \leq 0.003$). Therefore, we ignore the estimation error and assume the estimate MAF is the true MAF for each site.

2.2 The estimated effect size

With study sizes in the hundreds of thousands estimation error in MAFs is insignificant, but errors in estimating effect sizes are not. With study size $M \gg 1$, the estimated effect size, $\hat{\beta}$, as a function of the true effect size, β, at an allele of minor allele frequency x is

$$\hat{\beta}|\beta, x \sim N\left(\beta, \frac{1/M}{2x(1-x)}\right)$$

with effect sizes measured in units of the phenotypic standard deviation (V_P).

For a given selection coefficient, not only are the estimation errors approximately normally distributed but so is the true effect size

$$\beta \sim N\left(0, \frac{k}{4\mu} \cdot \frac{h^2}{L} \cdot s\right).$$

Therefore, estimated effect sizes at sites with selection coefficient s and MAF x are normally distributed as

$$\hat{\beta}|s, x \sim N\left(0, \frac{k}{4\mu} \cdot \frac{h^2}{L} \cdot s + \frac{1/M}{2x(1-x)}\right)$$

where the first term corresponds to the true effect and the second to the estimation error.

2.3 z-score

Since genome-wide significance is defined by a cutoff on the z-scores of variants, it is convenient to work with the z-scores instead of the estimated effect sizes, i.e. normalize the estimated effect sizes such that the error term is identically equal to 1. By this definition,

$$z = \sqrt{M \cdot 2x(1-x)} \cdot \hat{\beta}$$

and therefore

$$z|s, x \sim N\left(0, \frac{k}{4\mu} \cdot \frac{M \cdot h^2}{L} \cdot 2x(1-x) \cdot s + 1\right).$$

Note that, unlike the effect size, the z-score depends on both the selection coefficient and the minor allele frequency.

We define a combined GWAS power parameter

$$C = \frac{k}{4\mu} \cdot \frac{M \cdot h^2}{L}$$

which captures the scale of true signal (V_S/n or $\frac{k}{4\mu}V_G/L$) relative to the scale of estimation error (V_P/M). With this definition,

$$z|s, x \sim N\left(0, C \cdot 2x(1-x) \cdot s + 1\right).$$

It is this power parameter C, that we will directly infer in our inference. We denote the density function of the z-score as

$$P(z|C, s, x)$$

which is a normal distribution with mean zero and variance $C \cdot 2x(1-x) \cdot s + 1$.

2.4 Varying imputation quality

Ideally, the genotype at all sites would be perfectly estimated for all individuals. In practice, many sites are imputed and not directly genotyped. Even sites that are directly genotyped might be missing for some individuals. The result is that the study size, M, in the equations above varies between sites. We define the effective study size for site i as

$$M_i = \frac{1}{(\Delta\beta_i)^2 \cdot 2x(1-x)}$$

with $\Delta\beta_i$ being the reported standard error in effect size (usually estimated by resampling). We find the median study size over all SNPs with $x > 1\%$, including non-significant SNPs, as $M_{med} = \text{Median}(\{M_i\})$. M_{med} is nearly identical to the reported study size for each trait.

We define for each site a relative study size

$$m_i = \frac{M_i}{M_{med}}.$$

This relative study size is trait-independent and is proportional to (and essentially determined by) the info score that measures imputation quality. For included SNPs, m ranges from 0.8 to just over 1. m has a denoted dependency on the minor allele frequency and we estimate, for all SNPs, the distribution

$$P(m|x)$$

for different frequency bins (see bin definition in the next Section) using the standard errors for height effect sizes.

We now have

$$z|s, x \sim N\left(0, m \cdot C \cdot 2x(1-x) \cdot s + 1\right).$$

and the corresponding

$$P(z|m, C, s, x).$$

2.5 The joint distribution of MAFs and z-scores

We can now write expressions for the distributions of MAFs, z-scores among trait-affecting variants. Conditional on the selection coefficient, and power C, the distribution of MAFs and z-score, and effective study sizes is

$$P(x, z, m|C, s) = P(z|m, C, s, x) \cdot P(m|x) \cdot P(x|s)$$

and note that x and z are not independent, rather z is dependent on x. We can integrate over the effective study sizes to arrive at the distribution of MAF and z-scores for each selection coefficient

$$P(x, z|C, s) = \int_m P(z|m, C, s, x) \cdot P(m|x) \cdot P(x|s) \cdot dm$$

and lastly we can integrate over the selection coefficients to arrive at the overall distribution of x & z

$$P\left(x, z|C, f(s)\right) = \int_s P(x, z|C, s) \cdot f(s) ds.$$

We can take expectations over this distribution

$$E\left[g(x, z)|C, f(s)\right] = \int_x \int_z g(x, z) \cdot P\left(x, z|C, f(s)\right) \cdot dz \cdot dx.$$

and get expectation of quantities summed over all sites by

$$E\left[\sum_i g(x_i, z_i)|C, f(s)\right] = L \cdot E\left[g(x, z)|C, f(s)\right].$$

2.6 Threshold for genome-wide significance

We limit our inference to use GWAS hits, i.e. genome-wide significant variants. While it is difficult to estimate the MAF and effect size of causal effect sizes, there exist methods to (approximately) infer a set of SNPs representing independent signals such that each SNP should tag a single causal variant. Though such tagging SNPs are not themselves necessarily causal, they are in tight LD with the causal variants and therefore represent the causal variant's MAF and effect size.

A variant is considered genome-wide significant if its p-value under the null hypothesis of no effect size (null of $\beta = 0$) is below a threshold value, usually taken to be $5 \cdot 10^{-8}$. Since p-values are a monotonically decreasing function of the z-score squared, this threshold on the p-value translates to a threshold on the absolute value of the z-score. For a p-value threshold of $5 \cdot 10^{-8}$ this threshold is

$$|z| > z^* = \sqrt{2} \cdot \mathrm{erfc}^{-1}\left(5 \cdot 10^{-8}\right) \approx 5.45$$

with erfc^{-1} being the inverse complimentary error function (mercifully, we will not derive this formula here).

2.7 The conditional co-distribution of MAFs and z-scores

We now want to write an expression for the distribution of x and z for GWAS hits with MAF above 1%.

First, let's calculate the probability of a variant being a hit at $x > 1\%$ with relative study size m

$$Pr\left(hit|m, C, f(s)\right) = E\left[\mathbb{1}_{|z|>z^*} \cdot \mathbb{1}_{x>1\%}|m, C, f(s)\right] = \int_x \int_z \mathbb{1}_{|z|>z^*} \cdot \mathbb{1}_{x>1\%} \cdot P\left(x, z, m|C, f(s)\right) \cdot dz \cdot dx.$$

and therefore the conditional distribution of x, z and m is simply

$$P(x, z, m|hit, C, s) = \frac{P(x, z, m|C, s)}{Pr\left(hit|m, C, f(s)\right)}.$$

Note, that this distribution depends on the distribution of selection coefficients $f(s)$ and power parameter $C = M_{med} \frac{k}{4\mu} \frac{h^2}{L}$, but not on the target size L. The target sizes L determines the expected number of GWAS hits via

$$E[\#_{hits}|C, f(s)] = L \cdot Pr(hit|C, f(s))$$

with

$$Pr\left(hit|C, f(s)\right) = E\left[\mathbb{1}_{|z|>z^*} \cdot \mathbb{1}_{x>1\%}|C, f(s)\right] = \int_x \int_z \mathbb{1}_{|z|>z^*} \cdot \mathbb{1}_{x>1\%} \cdot P\left(x, z|C, f(s)\right) \cdot dz \cdot dx.$$

2.8 Estimating model parameters

We are finally ready to write down our likelihood and estimators for our model parameters.

Since the distribution of x and z depends on the distribution of selection coefficients $f(s)$ and power parameter $C = M_{med} \frac{k}{4\mu} \frac{h^2}{L}$, we can write a composite log-likelihood for these parameters as

$$LL\left(C, f(s)|\{x, z, m\}\right) = \sum_i \log\left(P\left(x_i, z_i, m_i|hit, C, f(s)\right)\right).$$

Our estimates $\hat{f}(s)$ & $\hat{C}$ are those that maximize this likelihood. Our estimate of h^2/L is

$$\frac{\hat{h^2}}{L} = E[s \cdot 2x(1-x)|\hat{C}, \hat{f}(s)]\frac{\hat{C}}{M_{med}} = \frac{4\mu}{k} \cdot \frac{\hat{C}}{M_{med}}.$$

The number of hits will be Poisson distribution with mean $L \cdot Pr(hit|C, f(s))$. Therefore, once we estimated $f(s)$ and C, the maximum likelihood estimator of L is simply

$$\hat{L} = \frac{\#_{hits}}{Pr(hit|\hat{C}, \hat{f}(s))}$$

with $Pr(hit|\hat{C}, \hat{f}(s))$ estimated using $\hat{f}(s)$ & $\hat{C}$.

2.9 Effects of joint effects size estimation on the standard error

Another small effect we incorporate is the fact that in the last step of COJO [3] effect sizes are estimated jointly for all hits using a reference panel. The size of the reference panel and the small LD between hits result in a slight change to the standard error in effect size estimation. This effects depends on the number of hits and patterns of LD between hits and is therefore, unlike the effects of imputations, different between different traits.

We estimate this effect for each SNP by dividing the square standard error in the original GWAS by that reported by COJO. We mark this term as d_i for hit i. d_i is usually close to, but slightly smaller, than 1 (see Fig. C). We incorporate it into our model a, per hit, small reduction in the study size. We therefore now replace C with $d_i \cdot C$ in our above equations, e.g. replace

$$P(x_i, z_i, m_i|hit, C, f(s))$$

with

$$P(x_i, z_i, m_i|hit, d_i \cdot C, f(s)).$$

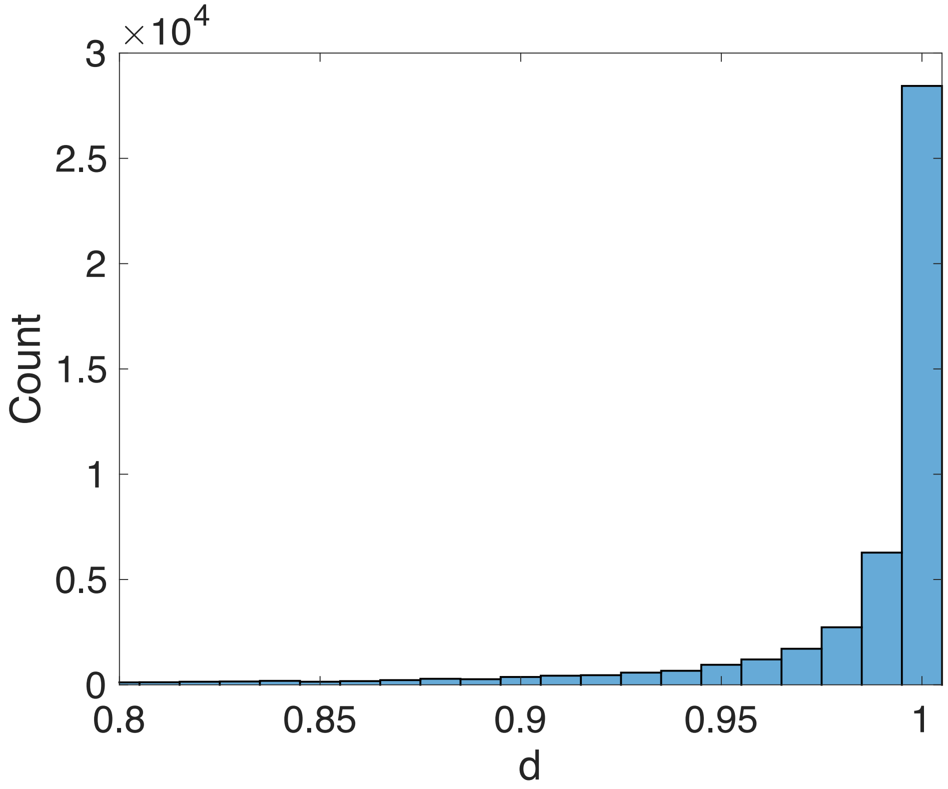

Figure C: **Histogram of d values for all GWAS hits used.** d values are very close to 1 for most hits. Figure data available at: https://doi.org/10.5281/zenodo.17041176.

2.10 How do MAFs and z-scores translate to selection coefficients?

It is not obvious from the above expressions for the likelihood how do the MAF and z-scores translate to selection coefficients. However, with a bit of analysis it is easy to see that, for each variant, the MAF provides an upper bound on the selection coefficient while the z-score provides a lower bound.

Let's think first of a population with a constant population size: When selection is as strong as (or stronger than) genetic drift, the MAFs of variants are mostly limited to MAFs below $1/2N_e s$, see Fig. DA. Therefore, the likelihood of a variant at a given MAF x coming for selection coefficient s, is flat for $s < 1/2N_e x$ and falls dramatically for larger selection coefficients, see Fig. DB. This same picture holds qualitatively for non-equilibrium demography too.

Under our model, z-scores squared are of the order of $C \cdot 2x(1-x) \cdot s$, i.e. larger for larger selection coefficients, see Fig. DC. This immediately suggests that the log-likelihood of s for a given z falls when s becomes being larger than $z^2/C \cdot 2x(1-x)$, as we can indeed see in Fig. DD.

Taken together, the MAF provides an upper bound on the selection coefficients and the z-score provides a lower bound, Fig. DE. Though these bounds still leave a lot of uncertainty for each variant, taken together even 100 variants are enough to estimate the distribution of selection coefficients.

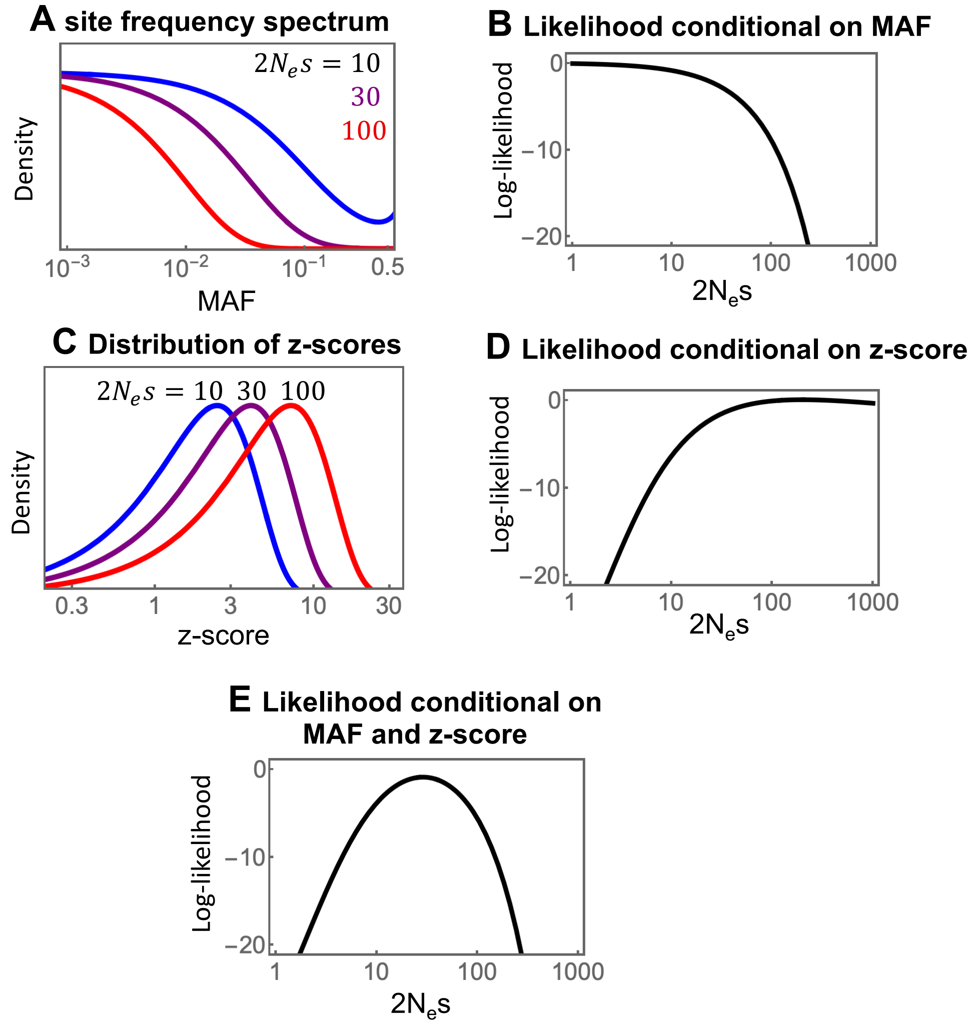

Figure D: **MAF and z-score bound the selection coefficient.** Illustration of how MAF and z-score bound the selection coefficient in a simplified scenario of constant population size and perfect knowledge of causal variants. **(A)** *The probability density of MAFs for different strengths of selection (the SFS).* ***(B)*** *The resulting log-likelihood of selection coefficients given a MAF of 10%.* ***(C)*** *The distribution of z-scores for different strengths of selection.* ***(D)*** *The resulting log-likelihood of selection coefficients given a z-score of 10.* ***(E)*** *The log-likelihood of selection coefficients given MAF of 10%* ***and*** *a z-score of 10. (We took $N_e = 10,000$ and $C = 5,000$.)*

3 Maximizing the likelihood

While writing down expressions of the likelihood is rather straightforward, reliably estimating this likelihood with a reasonable runtime has proven to be quite a challenge.

We then discuss a few practical hurdles to inferring our model's parameters. First, we explain how we estimate the likelihood in practice: We parametrize $f(s)$ as a log-spline and discretize some of the underlying parameters. Second, we explain our three-step approach to maximizing the likelihood and inferring the model's parameters: we find the best fitting $f(s)$ using MCMCMC (a Monte Carlo sampling approach similar to simulated annealing, see below). At each iteration, given $f(s)$, we perform a simple line search over h^2/L. Once $f(s)$ and h^2/L are estimated, we use those

estimates and the number of GWAS hits to estimate L. Lastly, we discuss the necessary addition of a regularization penalty on $f(s)$ to the likelihood.

3.1 Binning and gridding

In order to handle the calculations involved in estimating model parameters we treat x,s and C as discrete parameters. This allows us to formulate our equations as linear operations on probabilities. Since the z-score is normally distributed conditional on the other parameters, calculating the z-score's probability density is straightforward and we do not need to discretize it.

In order to bin the minor allele frequencies, we use 26 bins between 1% and 50%. The bin limits are:

$$\{10^{-2}, 10^{-1.9}, 10^{-1.8}, 10^{-1.7}, 10^{-1.6}, 10^{-1.5}, 10^{-1.4}, 10^{-1.3}, 10^{-1.2}, 10^{-1.1}, 0.1$$
$$, 0.125, 0.15, 0.175, 0.2, 0.225, 0.25, 0.275, 0.3, 0.325, 0.35, 0.375, 0.4, 0.425, 0.45, 0.475, 0.5\}$$

We look at selection coefficients on a dense grid on a log scale from $s = 10^{-8}$ to $s = 10^{0}$ in steps of $10^{1/16}$ (129 selection coefficients). We also consider the power parameter C on a dense grid on a log scale from $C = 10^{3}$ to $C = 10^{8}$ in steps of $10^{0.01}$ (501 C values).

3.2 Site frequency spectra

For each of the selection coefficients on our grid between 10^{-6} and 10^{-1} we ran our forward simulator (see details in Simons 2018 [1]) 120 million times, simulating 120Mbp of sites with that selection coefficient. We used a demographic model inferred using RELATE [4] on the British population(GBR) in the 1000 genomes dataset , see Fig. E. Since the distribution of common allele frequencies becomes insensitive to the selection coefficients when they are very small, we used the simulation for 10^{-6} when considering smaller selection coefficients (we consider such tiny selection coefficients in order to account for their possible effect on the distribution of effect sizes). Since no common variants appear for selection coefficients above 10^{-1} we just set the probability of seeing a hit from such selection coefficients as 0 (we consider such large selection coefficients in order to account for their possible contribution to heritability).

Thus, for each selection coefficient we estimate the proportion of variants with MAF above 1% and the distribution of their MAFs. This allows us to estimate, the probability of a variant being at each frequency bin, which we use as a proxy for $P(x|s)$.

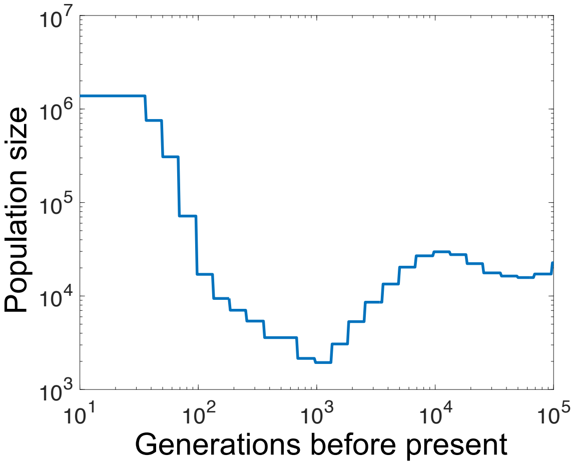

Figure E: **Demographic model.** *The demographic model we use was inferred for the GBR 1000 genomes population using RELATE [4]. Figure data available at: https://doi.org/10.5281/zenodo.17041176.*

3.3 Precaculated probabilities

Estimating our model parameters begins by precalculating all necessary probabilities conditional on s and C, for all s and C on our grid. For each GWAS hit i, we calculate the matrix

$$P^i_{C,s} \equiv P(x_i, z_i, m_i | d_i \cdot C, s) = P(z_i | m_i, d_i \cdot C, s, x_i) \cdot P(m_i | x_i) \cdot P(x_i | s).$$

This is a 501×113 matrix with three components:

- $P(z_i | m_i, d_i \cdot C, s, x_i)$ - the z-score probability. This is just a normal pdf with variance $m_i \cdot d_i \cdot C \cdot s \cdot 2x_i(1 - x_i) + 1$ estimated at z_i for each value of C and s.
- $P(m_i | x_i)$ - the probability of the relative study size. This is a single number that is independent of both C and s. This number will just add a constant to the log likelihood and therefore would not affect our parameter estimates. We therefore replace it with 1.
- $P(x_i | s)$ - the SFS of variants,. This is the probability of seeing a variant with selection coefficient s at MAF x, which we estimate using our forward simulations.

In addition, we precalculate the corresponding matrix of probabilities of variants being hits for all C and s,

$$P^{i,hit}_{C,s} = E\left[\mathbb{1}_{|z|>z^*} \cdot \mathbb{1}_{x>1\%} | d_i \cdot C, s\right] = \int_x \int_z \int_m \mathbb{1}_{|z|>z^*} \cdot \mathbb{1}_{x>1\%} \cdot P(z|m, d_i \cdot C, s, x) \cdot P(m|x) \cdot P(x|s) \cdot dm \cdot dz \cdot dx.$$

We numerically calculate these matrix monstrosities.

3.4 Calculating the likelihood via linear algebra

The advantage of precalculating all the probabilities, which takes an incredible amount of computation and memory, is that it is now extremely trivial to calculate the likelihood

$$LL\left(C, f(s) | x, z, m, d\right) = \sum_i \log\left(\frac{\sum_s P^i_{C,s} \cdot f_s}{\sum_s P^{i,hit}_{C,s} \cdot f_s}\right)$$

where we denote as f_s the column vector of size 129 representing $f(s)$ at each selection coefficient on our grid ($\sum_s f_s = 1$).

3.5 Estimating C for a given $f(s)$

It is now trivial to estimate C for a given $f(s)$. The likelihood conditional on $f(s)$ can be thought of as a row vector

$$LL(C) = \sum_i \log\left(\frac{\sum_s P^i_{C,s} \cdot f_s}{\sum_s P^{i,hit}_{C,s} \cdot f_s}\right)$$

and then

$$\hat{C} = \text{argmax}_C(LL(C))$$

and we denote the marginal log-likelihood at this estimate $\hat{C}$ as

$$LL\left(f(s)\right) = LL(\hat{C}) = \text{max}_C LL\left(C, f(s)|x, z, m, d\right).$$

3.6 Estimating $f(s)$ from the marginal likelihood

In principle, estimating $f(s)$ just involves maximizing $LL(f(s))$. In practice, in order to do that we need to somehow parametrize $f(s)$ with a parametrization that's both flexible and parsimonious. We do that via a log-spline, i.e. we parametrize the log of $f(s)$ as a spline, with s on a log scale.

The log-spline is parametrized by its value at specific knots, we use 5 knots, k, equally spaced (on a log scale) between $\log_{10} s = -8$ and $\log_{10} s = 0$, and our parameters are the value of $\log f(s)$ at $s = \{10^{k_i}\}$. Our $f(s)$ for every value of s is given by

$$\log f(s) = \text{spline}(\log_{10} s|\{k, \log f(10^k)\})$$

with spline here indicating a cubic spline from the 5 pairs $\{k, \log f(10^k)\}$ to $log_{10}s$. We estimate $f(s)$ at our 129 grid points to arrive at the vector f_s, which we normalize to 1. Because we lose a degree of freedom to normalization, the middle knot's value during the maximization process is set to 1 (pre normalization).

We then use Metropolis coupled MCMC (AKA, parallel tempering) [5] to find the values of $\{\log f(10^k)\}$ that maximize $LL(f(s))$, by sampling from $exp(T \cdot LL(f(s)))$ for large values of T. These values define our estimate $\hat{f}(s)$. In Fig. F of the main text, we can see these inferred distributions for all 95 traits (with TSDs).

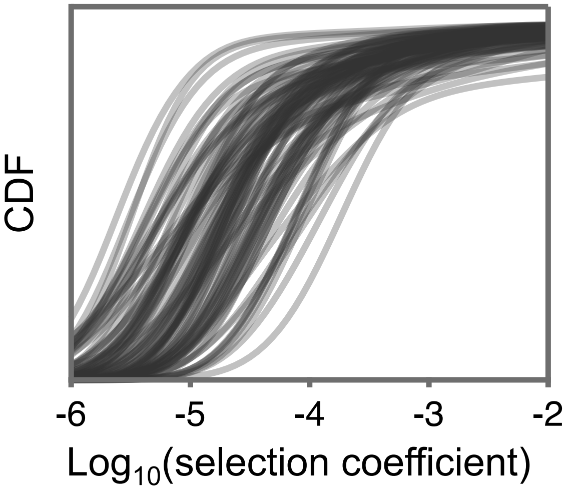

Figure F: **CDFs of the inferred distribution of selection coefficients for all 95 traits.** Presented here is the mean of the distribution over 100 bootstraps. Figure data available at: https://doi.org/10.5281/zenodo.17041176.

3.7 Maximizing the likelihood for a single shared distribution of s

So far, we have only considered one trait at a time, i.e. the trait-specific distributions (TSDs) model. Our setup is easily applicable to the single shared distribution model (SSD) too. For a given $f(s)$, we can find $\hat{C}_{trt}$ for each trait separately, exactly as shown above. We can then sum the resulting marginal log-likelihoods of each trait to arrive at a composite log-likelihood for $f(s)$ using all traits:

$$LL^{SSD}(f(s)) = LL^{trait\,1}(f(s)) + LL^{trait\,2}(f(s)) + LL^{trait\,3}(f(s)) + \cdots$$

We then maximize $LL^{SSD}(f(s))$ exactly as we would do for a single trait.
Note, that $f(s)$ is parametrized by four parameters. Therefore, for the TSD model we have 5 parameters per trait: C_{trt} plus four parameters for $f(s)$. For the SSD model we have four global parameters for $f(s)$ and one trait-specific parameter, C_{trt}. Thus, the SSD is vastly more parsimonious in the number of parameters it uses.

3.8 Adding a penalty to regularize $f(s)$

One complication we discovered is that this inference method misbehaves for very high or low selection coefficients since they do not contribute any GWAS hits. Very low selection coefficients have essentially no causal effect size, i.e. they only produce false positive hits. Very high selection coefficients don't produce common variants at all. Because we condition on variants being hits, the value of $f(s)$ at such extreme selection coefficients doesn't change our likelihood at all. Therefore, without additional information our inference procedure may (and does) assign arbitrarily large weight at these selection coefficients.

To counteract this tendency, we added a small penalty to the likelihood with two terms: one against having target sizes larger than the size of the genome and another against having heritabil-

ities larger than 1. The penalty takes the form

$$-\epsilon\left(\frac{\hat{L}}{3\cdot 10^8}+\frac{\hat{h^2}}{1}\right)$$

per each GWAS hit, where we calculate $\hat{L}$ and $\hat{h^2}$ using $\hat{C}$ and $\hat{f}(s)$ as described above. You can see in Fig. G how with low ϵ values of $f(s)$ explodes at high and low selection coefficients, while with high ϵ values of $f(s)$ becomes very narrow. Using our measure of model fit (see below) we find that $\epsilon = 0.05$ regularizes $f(s)$ while maintaining good fit.

3.9 Estimating confidence intervals

We estimate confidence intervals by block-wise bootstrap resampling. We use Berisa et al.'s division of the genome into 1702 approximately independent blocks [6]. We then resample, with replacement, 1702 blocks, and run the inference on the GWAS hits in the resampled blocks. We repeat this process 100 times to get a distribution the inferred parameters which we then use to estimate confidence intervals.

4 Estimating model fit

Having inferred a model from our data, we need to quantify how well the model fits the data, for both each trait and each variant. We introduce a measure that we call the residual p-value – which is a p-value with the inferred model as the null model estimated for each SNP. The residual p-value measures how effects are distributed relative to the model's prediction. We use the distribution of these p-values to estimate model fit to traits, identify outlier variants and set the value of our inference's single hyperparameter.

4.1 The residual p-value

By definition, the one-sided p-value for a measurement θ_i of a statistic θ is

$$p_i = Pr(\theta > \theta_i|\text{null})$$

where we know the distribution of θ under the null. By definition, p is uniformly distributed on the interval (0,1). Therefore, a low p-value allows us to reject the null for measurement i.

In GWAS, reported p-values can be thought of as one-sided p-values for the z-score square, z_i^2, under a null of no causal effect

$$p_i = Pr(z^2 > z_i^2|\text{null})$$

with z^2 having a chi-squared distribution with 1 d.f. under the null.

Our inferred model predicts the distribution of causal effects for variants of a given frequency and so we can define, for a variant of MAF x_i and z-score z_i, the residual p-value as

$$p_i = Pr(z^2 > z_i^2|C, f(s), x_i, m_i, d_i)$$

with C and $f(s)$ being our estimated model parameters, x_i the MAF and m_i the relative study size. However, our dataset includes only GWAS hits so we need to condition on z-scores being above

5.45. That's easily done under this framework with

$$p_i = \frac{Pr(z^2 > z_i^2|C, f(s), x_i, m_i, d_i)}{Pr(z^2 > 5.45^2|C, f(s), x_i, m_i, d_i)}.$$

If our model completely describes the co-distribution of MAF and z-score, then for GWAS hits this p-value should be uniformly distributed on the interval (0,1).

4.2 Cross-validated residual p-value

To avoid overfitting bias, we cannot estimate residual p-values on the same data on which we infer model parameters. Instead, we use Berisa et al.'s division of the genome into approximately independent blocks [6]. We then split the genome into 10 parts based on the last digit of the block number. This split ensures that the blocks in each part are well spaced from each other. For each of the 10 parts of the genome, we infer the model on GWAS hits in the other 9 parts. We use the inferred model parameters to calculate the residual p-values for GWAS hits on the held-out part. In this way, we estimate residual p-values for all GWAS hits.

4.3 Measure of model fit for a trait

If our model fits well, we expect the residual p-values for all GWAS hits for a trait to be uniformly distributed. If it does not, we expect to see some deviation. We use the Kolmogorov-Smirnov (KS) p-value for this distribution as our measure of model fit for a trait.
As one can see in Fig. 4 of the main text, these KS p-value are slightly smaller than expected under the null for both the TSD and SSD models. Looking at the KS p-values, no trait stands out as a clear outlier.
We therefore, use a Benjamini–Hochberg procedure to detect traits that deviate from the tested model with a false discovery rate of 0.05. That is, we order the KS p-values such that $p_{(1)}$ is the lowest p-value and $p_{(95)}$ is the highest p-value. We then find the highest k such that $p_{(k)} \leq 0.05 \cdot \frac{k}{95}$. Traits 1 to k are considered to deviate from the tested model with a false discovery rate of 0.05.
For TSD no trait is rejected.
For the Alpha model all traits were rejected except for two: Age first had sexual intercourse, and Peak expiratory flow (PEF).
For SSD 27 trait are rejected: Glycated haemoglobin, Mean platelet (thrombocyte) volume, High light scatter reticulocyte count, Platelet distribution width, Mean sphered cell volume, Triglycerides, SHBG, Impedance of leg (left), Mean reticulocyte volume, Alkaline phosphatase, High light scatter reticulocyte percentage, Gamma glutamyltransferase, Reticulocyte percentage, Haemoglobin concentration, Platelet count, Red blood cell (erythrocyte) distribution width, Arm fat percentage, Mean corpuscular haemoglobin, Trunk fat percentage, Mean corpuscular volume, Monocyte count, Waist circumference, Body fat percentage, Monocyte percentage, Platelet crit, Impedance of whole body, Weight.
See Supplementary Table S1 in a separate csv file.

4.4 Measure of model fit for variants

We want to identify outlier variants, since they are potentially important to understanding the biology and/or evolution of a trait. We define an outlier p-value as one with

$$p < \frac{0.05}{n_t}$$

with n_t being the number of hits for the trait. This criterion is just an 0.05 threshold with a Bonferroni correction.

We find 30 such outlier hits, though some of them are outliers for more than one trait.

4.5 Accounting for outlier variants during inference

Outliers are common with large z-scores in the range of 50-100 We find 56 outliers, but since some of them are the same outlier affecting multiple traits, we have 30 unique outliers. With 47,011 hits, this suggests approximately 1 in every 1000 hits is an outlier. We used these observations to introduced a term into the likelihood to capture those outlier hits and prevent them from biasing our results: We assume that outliers have frequencies similar to neutral sites, so $P(x|outlier) = P(x|s = 10^{-6})$, and their z-scores are normally distributed with mean zero and std 100, meaning that $P(z|outlier) = \frac{1}{100\sqrt{2\pi}} \exp(-z^2/200)$. We then condition on outlier being hits

$$P(x, z|outlier\, hit) = \frac{P(x|outlier)P(z|outlier)}{E[\mathbb{1}_{|z|>z^*} \cdot \mathbb{1}_{x>1\%}|outlier]}.$$

Lastly, we then assume that 1/1000 hits are outlier so

$$P\left(x_i, z_i, m_i|hit, d_i \cdot C, f(s)\right)$$

becomes

$$\frac{999}{1000} \cdot P\left(x_i, z_i, m_i|hit, d_i \cdot C, f(s)\right) + \frac{1}{1000} \cdot P(x_i, z_i|outlier\, hit).$$

We run our inference with these modified probabilities and reestimate model parameters and see that we get consistent results. All results presented from the main text are for the inference with this control for outliers.

After we estimate model parameters we can estimate the probability that a variant is an outlier as

$$P\left(outlier|x_i, z_i, m_i\right) = \frac{\frac{1}{1000} \cdot P(x_i, z_i|outlier\, hit)}{\frac{999}{1000} \cdot P\left(x_i, z_i, m_i|hit, d_i \cdot C, f(s)\right) + \frac{1}{1000} \cdot P(x_i, z_i|outlier\, hit)}.$$

and exclude variants from our analyses of model fit per trait if $P\left(outlier|x_i, z_i, m_i\right) > 0.5$ for at least 5 of the jackknife parameter estimates. Unsurprisingly, all 15 excluded variants are also outlier variants in the previous analysis.

4.6 Residual p-values for alternative models

We wanted to test the fit of other models to data. We therefore repeated the same procedure, just with other models. We fit a maximum likelihood model where effect sizes are normally distributed with mean zero and variance $A \cdot (x(1-x))^{\alpha}$, with A and α being the model parameters. When we set $\alpha = 0$ and only infer A, we get a simple normal distribution and when we infer the value of α this is the “alpha model”. We again infer the model on 90% of the genome and calculate residual p-values (conditional on genome-wide significance) for the other 10%.

4.7 Tuning ϵ

We use the residual p-values to tune the hyperparameter controlling the strength of the penalty in our likelihood, ϵ. We tried different values of ϵ, and saw that when $\epsilon <= 0.001$ we get unrealistic target sizes and heritabilities. On the other hand, for $\epsilon >= 0.01$ the Kolmogorov-Smirnov p-values for model fit for traits become very small, suggesting such large penalty affects model fit. We therefore chose $\epsilon = 0.005$, for this hyperparameter. See Fig. G.

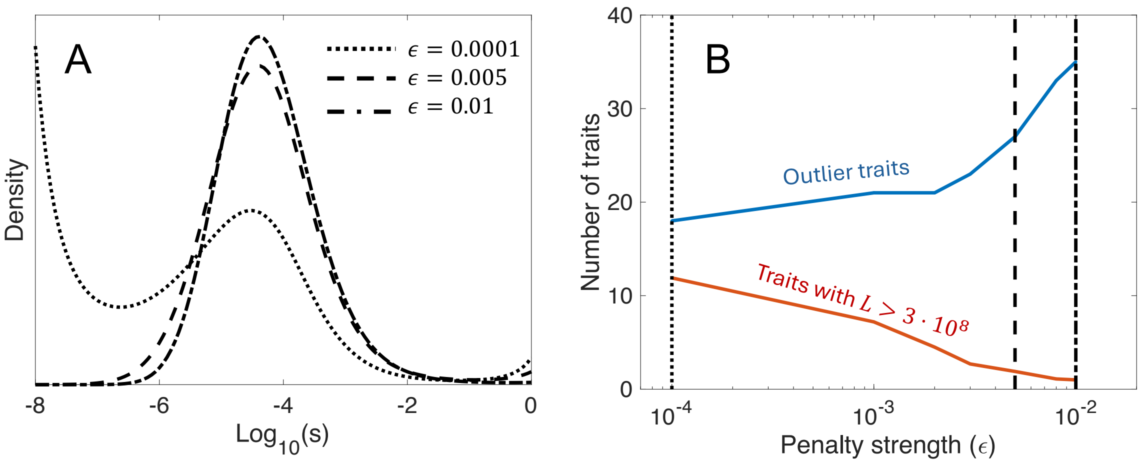

Figure G: **Tuning the strength of the penalty.** *(A) If the penalty is too weak, we get spurious weight on the high and (mainly) low selection coefficients. (B) If the penalty is too strong, the model stops fitting the data. If the penalty is too weak, the estimated target sizes are unrealistic. Figure data available at: https://doi.org/10.5281/zenodo.17041176.*

4.8 Choosing the number of knots

The number of knots in out log-spline parametrization of $f(s)$ controls the distribution's smoothness. Each additional knot allows for more flexibility in the shape of $f(s)$. We tested different numbers of knots uniformly positioned between $log_{10}s = -8$ and $log_{10}s = 0$. As you can see in H, we get the best model fit for 5 knots, which is indeed what we use throughout.

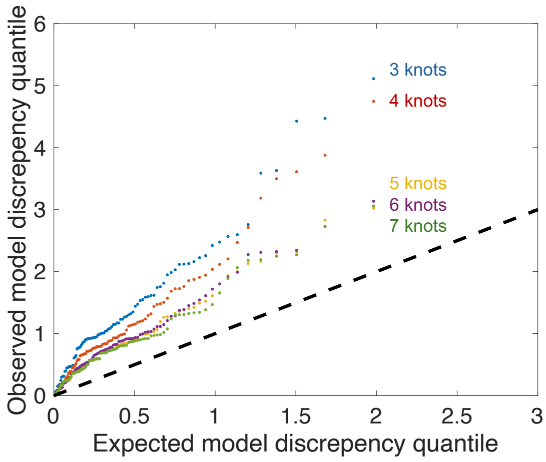

Figure H: **Choosing the number of knots.** *We look at TSD model discrepancy with different numbers of knots and see that 4 knots provide the best model fit. The same picture emerges for SSD, but the differences are much more subtle. Figure data available at: https://doi.org/10.5281/zenodo.17041176.*

4.9 Consistency with lower sample sizes

To show that the inference is consistent across different sample sizes, we used subsets of the UK biobank to 50,000, 100,000 and 200,000 individuals and ran a GWAS for height for each. Note, that each subset is chosen from the previous subset, such that the 50,000 individuals are all included in the 100,000 individuals. This scheme is supposed to simulate a gradual increase in sample size (rather than independent GWAS). As you can see in Fig. I, inference results are consistent across sample sizes.

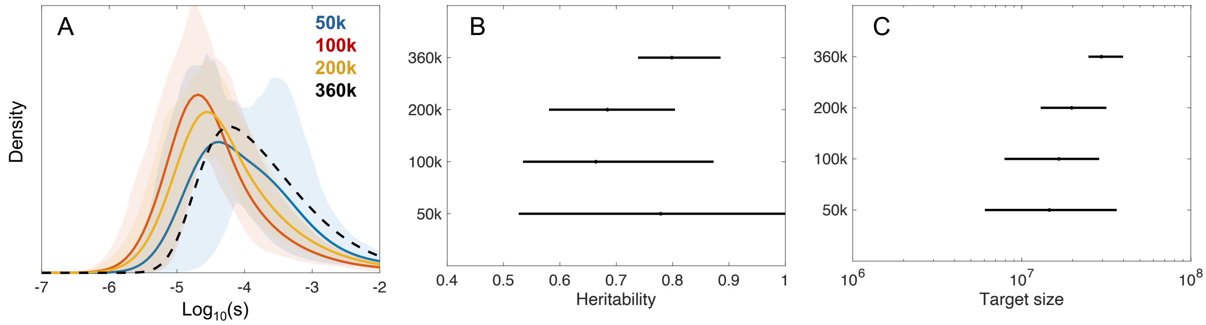

Figure I: **Lowered sample sizes.** *We look at TSD model results for height with different sample sizes. We see that the distribution of selection coefficients (A), and the point estimates of target size (B) and heritability (C) are all consistent across sample sizes. Figure data available at: https://doi.org/10.5281/zenodo.17041176.*

4.10 Consistency with twin and SNP heritabilities

Our SSD point estimates for heritability are broadly consistent with twin and SNP heritabilites. We used SNP heritabilities from the Neale lab dataset and searched the literature for estimates of twin heritability for 9 traits. As you can see in Fig. J, our heritability estimates tend to fall between SNP and twin heritability.

Note that the confidence intervals on our hertiability estimates (Fig. 3, Table S1) are much greater than those for SNP heritability because they rely only on GWAS hits.

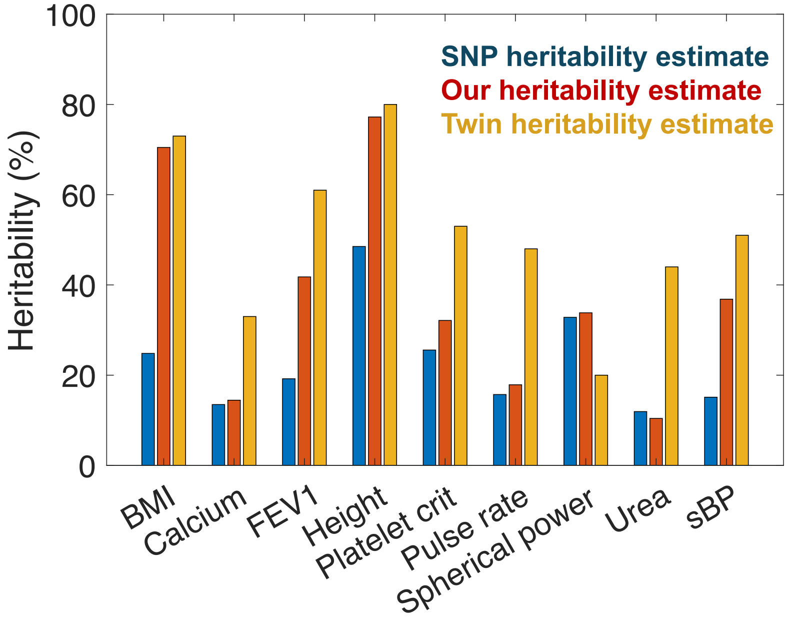

Figure J: **Heritability estimates.** *We compare SNP heritability estimates (in blue), our SSD point heritability estimates (in red), and twin heritability estimates (in orange). Twin heritability estimates citations: BMI [7] , Calcium [8] , FEV1 [9], height [10], Platelet crit [11], Pulse rate [12], Spherical power [13], Urea [14], sBP [15]. Figure data available at: https://doi.org/10.5281/zenodo.17041176.*

4.11 Contribution of rare variants

Our analysis is inherently limited in its ability to understand the contribution of rare variants and/or variants with large selection coefficients to the genetic architecture. It is therefore difficult to directly comment on whether similarities in genetic architecture extend to rare variants. However, Pathan et al. [16] have recently estimated the contribution of rare variants to the heritability of a wide array of complex traits. Under our model, this quantity depends on $f(s)$ through

$$\frac{h^2_{rare}}{h^2_{common}} = \frac{\int_{x<1\%}\int_\beta 2\beta^2 x(1-x)P(x,\beta|s)f(s)}{\int_{x>1\%}\int_\beta 2\beta^2 x(1-x)P(x,\beta|s)f(s)} = \frac{\int_{x<1\%} s\cdot x(1-x)P(x|s)f(s)}{\int_{x>1\%} s\cdot x(1-x)P(x|s)f(s)}$$

and would therefore be similar for traits with similar $f(s)$. As you can see in Fig. K, the proportion of variance due to rare variants is similar across traits suggesting that the similarity we observe for common variants may extend to rare variants for most traits.

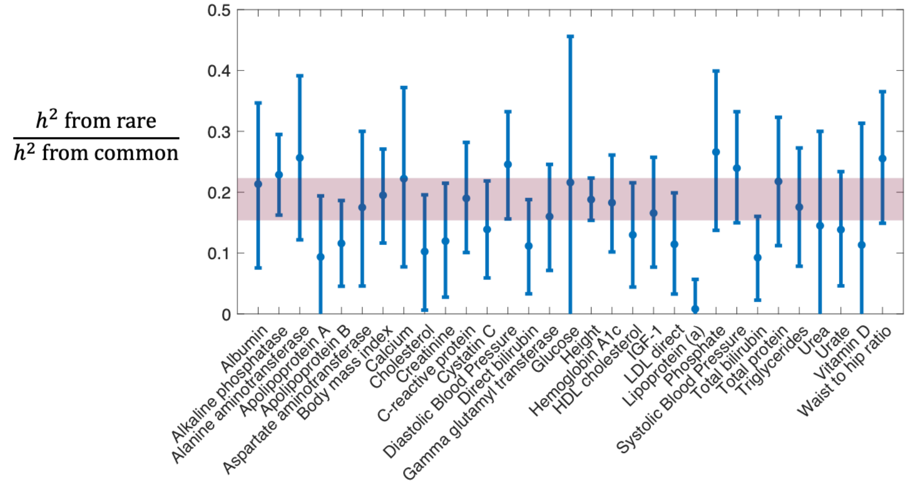

Figure K: **Proportion of rare variants.** *Heritability from rare variants divided by heritability from common variants as estimated in Pathan et al. (Table 1 in [16]), for each trait we see the point estimate and 95% CI. The 95% CI for height are highlighted in pink and we can see that CIs of all traits except "Lipoprotein (a)" overlap with the CIs for height. This suggests that the similarity between traits extends also to rare variants. (We neglected the relatively small uncertainty in common variant heritability estimates so the CIs are slightly underestimated.)*

4.12 TSD vs. SSD fit for a trait

To evaluate how well the SSD model applies across all 95 traits, we employ the likelihood ratio test for nested models. The idea behind the test is that if we fit our data with two models, a simple model and a complex model, with the complex model having j additional parameters then if the two models fit equally well then

$$2 \cdot \log \frac{L_{complex}}{L_{simple}}$$

has a chi-squared distribution with j degrees of freedom. If this values is largerr than expected, that's evidence that the more complex model is well-justified by data.

Using the TSD as the complex model and the SSD as the simple model, with the SSD having 4 extra parameters per trait, we reject 50 traits with at a false discovery rate of 0.05. This suggests that the TSD is well-supported for about half of the traits.

5 Validating our inference using simulations

In order to test our inference, we simulate data from our model and run the inference on it. We use both the SSD model and TSDs. We see that the inference capture the distribution of medium-range selection coefficients ($-6 < log_{10}(s) < -2$) very well, since they produce GWAS hits. However, contributions to trait architecture from beyond this range are missed, meaning that we may underestimate target sizes and heritabilities. Lastly, we show that if a dataset contains a mixture of traits from different distributions of selection coefficients, then TSDs are a much better fit to the data than the SSD, as expected.

5.1 Simulating a single trait

Given the parameters L, h^2 and $f(s)$, and in addition study size M, we want to simulate a set of hits based on our model with a set of MAFs, z-scores and relative study sizes $\{x_i, z_i, m_i\}$ (we set $d_i = 1$). We use the following algorithm:

1. Set $C = M \cdot \frac{h^2}{L} \frac{1}{E[2q(1-q) \cdot s | f(s)]}$.
2. Calculate the probability of a variant being common $P(common) = \sum_s P(x > 1\%)|s) \cdot f(s)$.
3. Draw the number of common causal variants from a binomial distribution with L tries and probability $P(common)$.
4. Draw the number of these causal variants with each selection coefficient from a multinomial distribution with L tries and probabilities $P(x > 1\%)|s) \cdot f(s)/P(common)$.
5. For each the causal variants with a selection coefficient s, draw x_i from $P(x|s)$ conditional on $x > 1\%$.
6. Draw m_i (and the corresponding LD-score) with replacement from the m_i at the frequency bin corresponding to x_i for each variant.
7. Draw z_i from the distribution $N(0, C \cdot m_i \cdot s_i \cdot 2x_i(1 - x_i) + 1)$.
8. Keep only hits, i.e. variants with $|z_i| > 5.45$.

5.2 Simulating sets of traits

We simulate sets of traits, with traits having one of the following 4 specified distributions of selection coefficients (see Fig. L):

1. $log_{10}(s) \sim N(-4, 0.5)$
2. $log_{10}(s) \sim N(-3, 0.5)$
3. $log_{10}(s) \sim N(-2, 0.5)$
4. $s \sim \Gamma(0.1, 3 \cdot 10^{-2})$

In each set, we simulate 25 traits with each possible pair from heritabilities $(0.1, 0.25, 0.5, 0.75, 0.9)$ and target sizes $(3 \cdot 10^6, 10^7, 3 \cdot 10^7, 10^8, 3 \cdot 10^8)$. We run both the TSD and SSD inference on each of these 25 traits separately, as well as on all of the 100 traits in tandem.

5.3 Validating our inference on simulated traits

As you can see in Fig. LA, with 25 traits the SSD model captures the distribution of selection coefficients extremely well. However, when the distribution includes significant contributions from high ($log_{10}(s) > -2$) or low ($log_{10}(s) < -6$) selection coefficients, our inference truncates the distribution. Since these extreme selection coefficients do not produce any GWAS hits, our inference puts no weight on them (because we have a parsimony-inducing penalty on our likelihood). Therefore, the distribution of selection coefficients we infer is the distribution at the medium range of

selection coefficients ($-6 < log_{10}(s) < -2$). Our inference only estimates the heritability and target size from within this medium range of selection coefficient. Therefore, for distributions of selection coefficients which are entirely within this range, our inference provides an unbiased estimates of the entire heritability and target size, Fig. LB&C. However, for distributions with contributions from outside this range, our inference systematically underestimates the heritability and target size by a constant.

As expected, the SSD and TSD have similar levels of discrepancy between inferred model and data, Fig. LD. However, when we simulate a dataset with 25 traits from each of the 4 distributions and infer a SSD on it, we see that the inferred distribution only approximates 1 out of the 4 distribution, i.e. it misspecifies the distribution for most traits, Fig. MA. Unsurprisingly, under this scenario, there is huge discrepancy between the SSD model and the data, which is completely absent from the TSD model, Fig. MB. Note how this discrepancy is much smaller for distribution 2, which is similar to the inferred SSD. Compare this result with Fig. 4C of the main text, where we do not see such a difference between TSD and SSD in our data.

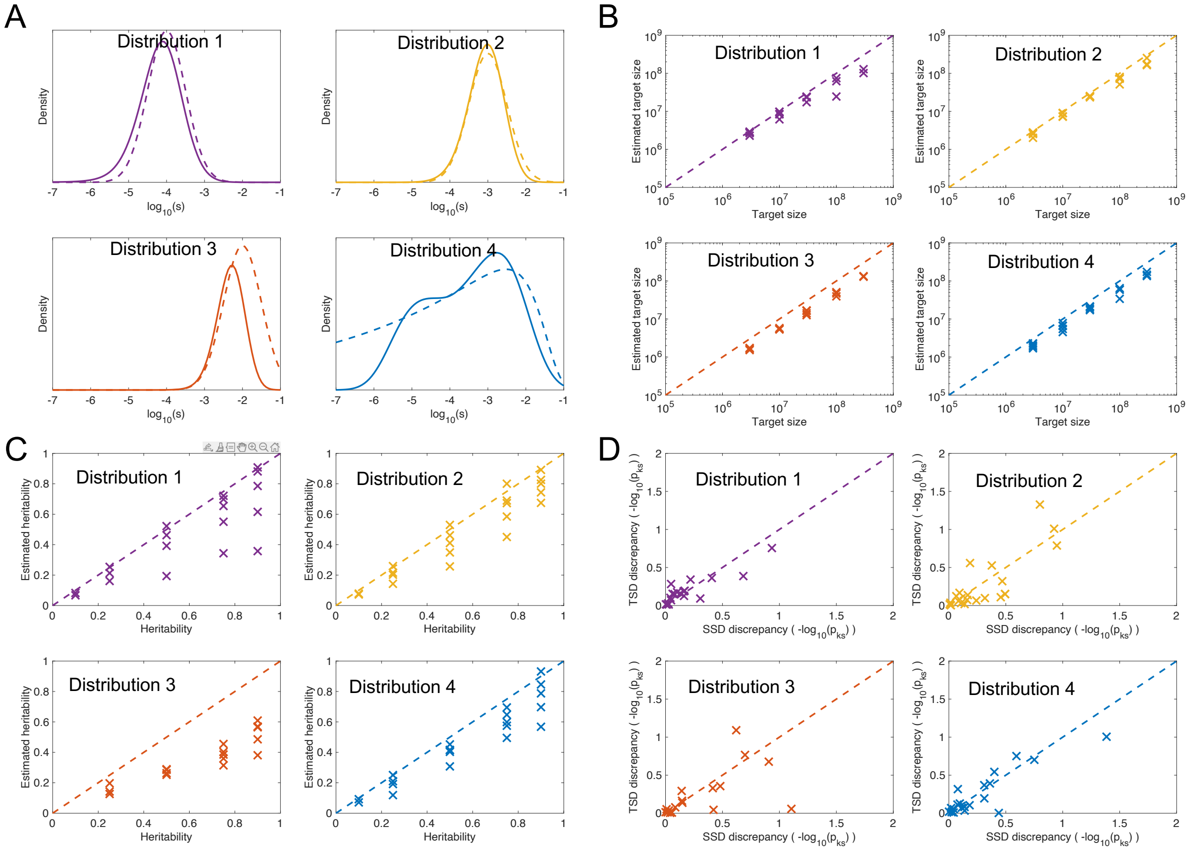

Figure L: **Inference on simulated datasets.** **(A)** *The distribution of selection coefficients (normalized to 1 in the range of* $-6 < log_{10}(s) < -2$*) is well captured by the SSD inference. True distributions in dashed lines, inferred distribution in continuous.* ***(B-C)*** *Estimates of target size and heritability correlate very well with true target size and heritability.* ***(D)*** *TSD and SSD have low and similar discrepancy to data. Figure data available at: https://doi.org/10.5281/zenodo.17041176.*

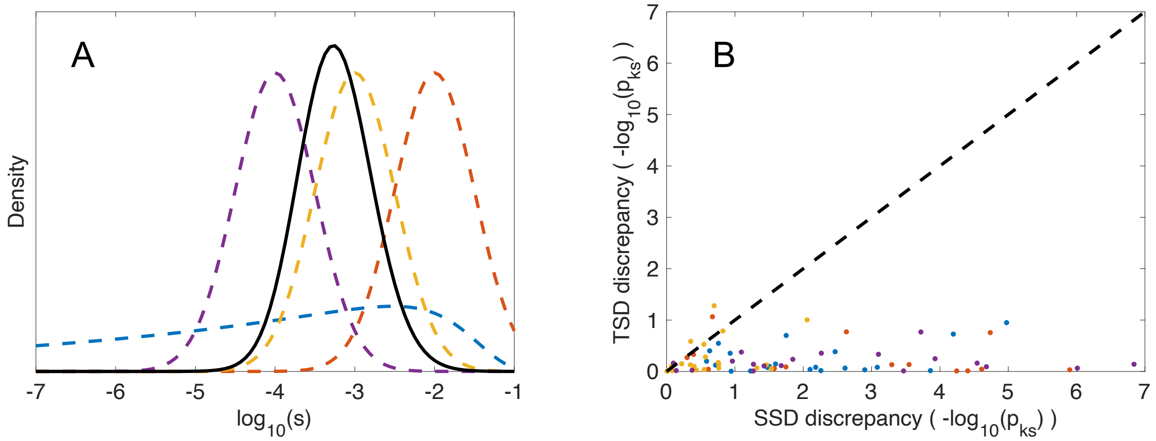

Figure M: **Inference on a non-SSD dataset.** *Inference on a dataset of 100 traits, 25 from each of 4 different distributions of selection coefficients.* ***(A)*** *The inferred distribution of selection coefficients under a SSD model can match, at most, one of the underlying distributions. True distributions in dashed lines, inferred distribution in a black continuous line.* ***(B)*** *The SSD model has large discrepancy with data, while the TSD model does not (datapoints colored by underlying selection coefficient distribution). Figure data available at: https://doi.org/10.5281/zenodo.17041176.*

6 UKBB Dataset

In our inference, we use GWAS hits from 95 continuous traits from the UK biobank. Here we describe the dataset, variant filtering and trait choice.

6.1 GWAS summary statistics for diverse traits

The Neale lab performed standardized GWAS on all available UK biobank phenotypes (`http://www.nealelab.is/uk-biobank`) and we use their summary statistics (version 3). We chose to focus only on continuous phenotypes, using GWAS results based on both sexes and inverse rank-normal transformed ("irnt") phenotypic values. We will address categorical phenotypes and disease risk in future work.

6.2 Trait choice

Based on simulations, we were aiming to keep traits with at least 100 hits. In addition, we wanted to minimize trait repetition and avoid traits whose genetic architecture is concentrated at a single locus.

For trait screening we used PLINK's LD-based clumping [17]. For each trait in the GWAS data, we used plink's –clump flag with a p-value threshold of $5 \cdot 10^{-8}$, LD threshold of $r^2 = 0.1$ and physical distance threshold of 1Mb. We only kept traits with at least 100 clumped hits at MAF above 1%. This left us with 138 traits.

Next, if two traits are identical except for handedness, e.g. Arm fat percentage (left) and Arm fat percentage (right), we kept the one with the (slightly) larger number of hits. This left us with 114 traits.

Lastly, we counted the number of hits at each approximately-independent genomic block from [6]. If over 10% of hits reside in a single genomic block (out of 1702 blocks) we dropped the trait. At the end of this process, we were left with 96 traits.

6.3 Variant filtering

Starting with the set of 13.7 million variants ascertained by the Neale lab for GWAS ("imputed-v3 Variant QC"), we restricted our analysis to variants passing the following filters: (i) autosomal, (ii) bi-allelic (genomic positions where at most two alleles had frequency > 0.001), and (iii) MAF $> 1\%$.

Next, we ran COJO [3] on each trait to arrive at approximately independent hits and co-estimate their effect sizes. We used the parameters –cojo-p 10^{-6} and –cojo-slct. As a reference panel for LD, we used 10000 randomly chosen individuals from the UKBB, passing the following QC measures: (i) reported gender matched the inferred sex from genotype data, (ii) were not heterozygosity outliers, (iii) did not have excessive number of relatives, and (iv) did not carry sex chromosome aneuploidies. We further restricted the panel to individuals labeled by the UKBB team to be "White British" and were chosen for principal component analysis (PCA) to exclude close relatives.

We filtered out variants with LD score above 500 or INFO score below 0.8. After filtering, the number of hits for one of the traits dropped below 100 and so we were left with the 95 traits in Supplementary Table S1.

6.4 Subset of independent traits

In order to test the extent to which our SSD result are influenced by dependencies between traits, we also ran the inference on a subset of traits chosen to be approximately independent. Independent traits were chosen using the following protocol:
(1) Start with list of all traits L_{all}, and an empty list of chosen traits L_{choice}.
(2) Choose trait with most hits in L_{all}, add it to L_{choice} and remove it from L_{all}.
(3) Remove all traits with $|r_g| > 0.2$ with the chosen trait from L_{all} (genetic correlation r_g estimates taken from the Neale lab analysis).
(4) If L_{all} is not empty, go back to (2).
(5) Done.

The resulting list consists of 15 traits which are genetically nearly independent. We inferred the parameters of the SSD model on this subset of traits, and as can be seen in Fig. N got results that are essentially identical to the SSD model on the full dataset. We therefore conclude that dependencies between traits have only a small influence on our SSD results, if any.

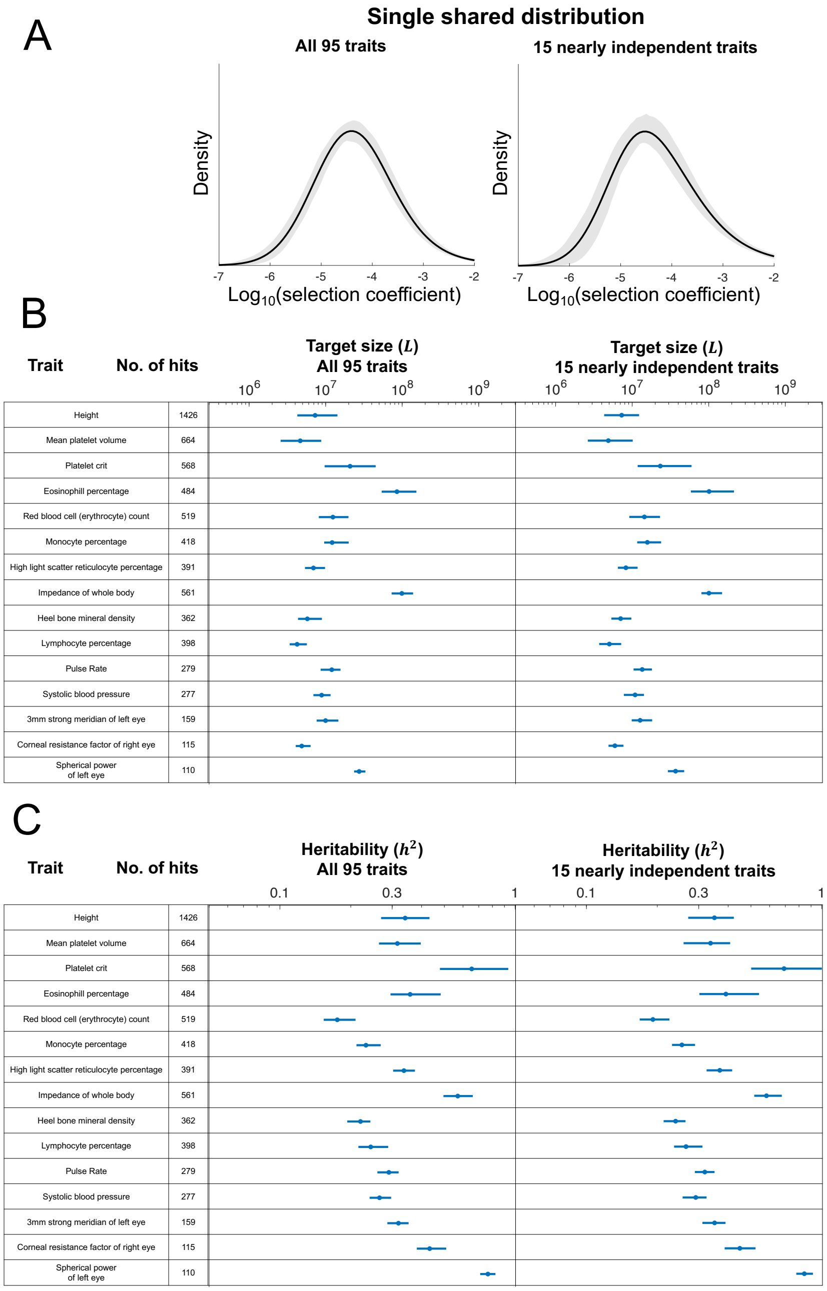

Figure N: **SSD results for 15 nearly independent traits.** *(A) The SSD is nearly identical for the full dataset and the subset of 15 nearly independent traits. (B) Accordingly, target size estimates are highly consistent for those 15 traits. (C) Estimates of heritability are highly consistent too. Figure data available at: https://doi.org/10.5281/zenodo.17041176.*

6.5 Summaries of GWAS hits

The GWAS hits for the 95 traits differ in their number, effect sizes and minor allele frequencies, as seen in Fig. O. Despite restricting ourselves to traits with at least 100 hits, the number of hits still spans an order of magnitude (Fig. OA). These hits vary in their z-scores, and the magnitude of z-scores doesn't have any clear relation to the number of hits (Fig. OA). However, the mean

MAF is very similar across all trait (Fig. OB), which might be the result of restricting ourselves only to common variants. Since the number of hits and their effect on traits vary between traits, the proportion of phenotypic variance explained by the hits for each trait varies greatly (Fig. OC).

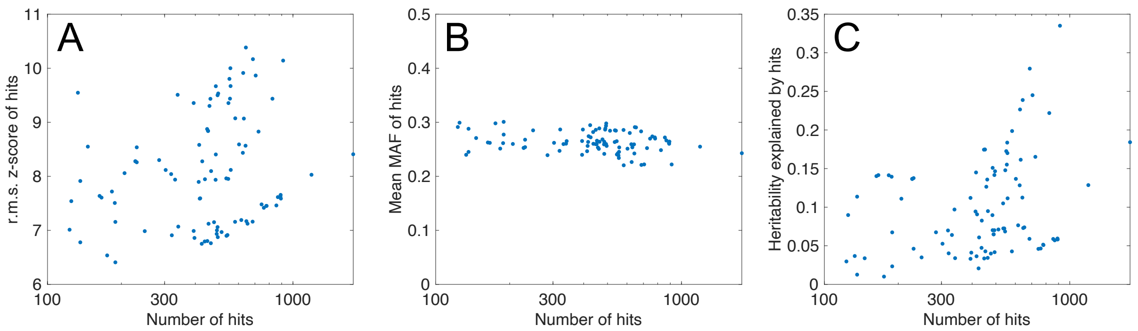

Figure O: **Summaries of GWAS hits for the 95 traits.** *(A) The root mean squared z-score differs between traits and is not determined by the number of hits, which spans an order of magnitude. (B) In contrast, the mean MAF is very similar across all traits. (C) The proportion of phenotypic variance explained by hits also greatly differs between traits. Figure data available at: https://doi.org/10.5281/zenodo.17041176.*

6.6 Functional followup of outlier variants

15 variants in our dataset are clear outliers, having been detected as outliers in the inference (see Section 4.2) and via low residual p-values (see Section 4.4). In order to understand which genes they affect we annotated each SNP's gene and variant type using dbSNP. The majority of variants were within gene boundaries of a known gene and were annotated with that gene. Two variants (rs700750 and rs386698213) were not within a gene window and thus were annotated as noncoding variants. We annotated these noncoding variant's with their nearest genes using a list of all genes in any GO, KEGG, or Reactome MSigDB pathway (in order to avoid pseudogenes and genes of unknown function) and manual curation using GeneCards to prioritize a single gene per SNP. GeneCards was also used to identify each gene's known function for all 16 variants. GWAS catalog was used to identify each SNP's relevant trait and disease associations. These results are presented in Supplementary Table S2 where candidate mechanisms for how each variant affects their respective gene, protein, and the trait are listed in column 11.

6.7 Computational reduction in study size

When comparing the genetic architecture of traits, we want to equate their median study sizes. In order to so, we take the output of COJO, which has a p-value threshold of 10^{-6}. We introduce additional Gaussian noise to each z-score:

$$z_{330k} = \sqrt{\frac{330,000}{M_{med}}} \cdot z + \sqrt{1 - \frac{330,000}{M_{med}}} \cdot \epsilon$$

with M_{med} being the median study size for the trait and ϵ drawn from a Normal distribution with mean 0 and variance 1.

7 Are GWAS hits a good proxy for the underlying causal variants?

Our analysis relies on genome-wide significant associations (GWAS hits), which tag the underlying causal variants but may not affect the trait themselves. Our analysis assumes that the number GWAS hits, their minor allele frequencies and effect sizes as estimated by COJO are a good proxy for the number of underlying genome-wide significant causal variants, their minor allele frequencies and effect sizes. In order to understand if this assumption is valid we break it down into four questions that we address sequentially using simulations and data:

7.1 How does the choice of tagging variants affect our estimates of frequency and effect size?

GWAS hits tag causal variants which affect a trait. Most causal variants have multiple variants in high correlation (due to strong LD) which can tag them. The choice of tagging variant is an additional source of noise which is not captured by our model. We show using a dataset of fine-mapped GWAS signals for a multitude of UK biobank traits, that this effect is negligible because variants can be highly correlated only if they're extremely close in their minor allele frequency.

7.1.1 Fine-mapped traits in the UK biobank

We use a dataset of 94 traits (partially overlapping our 95 traits) in the UK biobank [18] that were fine-mapped using SUSIE [19]. Fine-mapping is a Bayesian approach used to statistically identify causal variants from GWAS. For each GWAS signal it produces a "credible set" - a group of variants that have at least 95% probability to include the causal variant. Within a credible set, such a method gives the probability that each variant is the causal variant (probability of inclusion). In order to quantify the uncertainty in the frequency and effect of the causal variant underlying a credible set we define a weight for each variant within a set

$$w_i \propto \mathrm{PIP}_i$$
$$\sum_i w_i = 1$$

with i indexing the variants within the set. We then quantify the uncertainty within a set by weighted sums over the weights. If x_i is the minor allele frequency of variant i and z_i its the $z - score$ for the trait increasing allele of this variant, then we define

$$\bar{x} = \sum_i w_i x_i$$
$$\bar{z} = \sum_i w_i z_i$$

and

$$\Delta x = \sqrt{\sum_i w_i (x_i - \bar{x})^2}$$
$$\Delta z = \sqrt{\sum_i w_i (z_i - \bar{z})^2}.$$

This allows us to define the relative uncertainty in x and z as $\Delta x/x$ and $\Delta z/z$ due to choice of tagging variant and see that it is very small. As we can see in PA the relative uncertainty in minor allele frequency is small, with a median uncertainty of 1.5% and the uncertainty being below 10% for 90% of sets. Similarly, we can see in PB the relative uncertainty in z-score is extremely small, with a median uncertainty of 1% and the uncertainty being below 3% fro 90% of sets. Also note, that for about 15% of sets there is no uncertainty in the causal variant at all. These results suggest that the uncertainty in minor allele frequency and effect size introduced by choice of tagging variant is relatively small (about 1% and less than 10%).

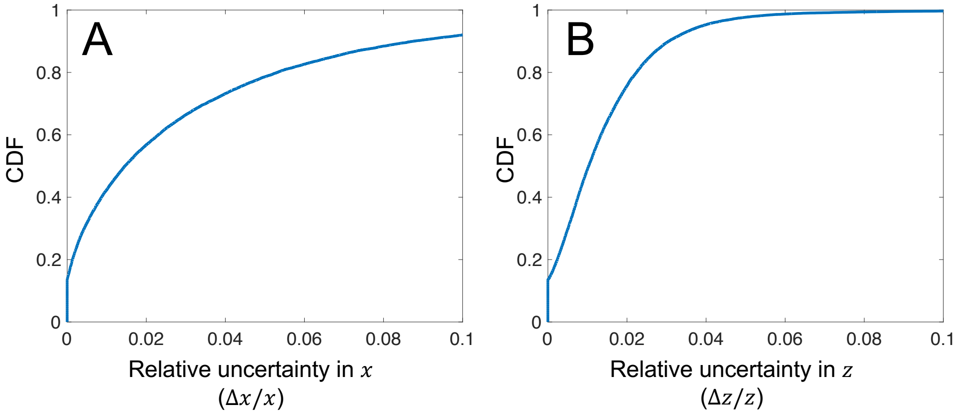

Figure P: **Relative Uncertainty.** *The CDF of the relative uncertainty induced by choice of tagging variant within a credible set for* ***(A)*** *minor allele frequency and* ***(B)*** *z-score. Results shown for sets with* $\bar{x} > 1\%$ *and* $\bar{z} > z^* \approx 5.45$. *Figure data available at: https://doi.org/10.5281/zenodo.17041176.*

7.1.2 The reason choice of tagging variants is a small effect

It might seem surprising the choice of tagging variants has only a small effects on estimates of minor allele frequency and effect size. However, there is an intuitive theoretical explanation.
If x_a and x_b are the minor allele frequencies of variants a and b then the maximal r^2 for the correlation between their genotype is [20]

$$r^2_{\text{max}} = \max\left(\frac{x_a/(1-x_a)}{x_b/(1-x_b)}, \frac{x_b/(1-x_b)}{x_a/(1-x_a}\right).$$

Therefore, $r^2_{\text{max}} = 1$ if and only if $x_a = x_b$, otherwise it tapers off quickly as x_a and x_b diverge from each other (Fig. Q).
For there to be an uncertainty about the causal site, variants have to be at high LD. Since high LD is only possible when minor allele frequencies are very close, there should be only small uncertainty in minor allele frequency within a credible set. Because minor allele frequencies are similar and variants are highly correlated, there is also only little uncertainty in the z-score.
Note, however, that is entirely possible for variants to have differing derived allele frequency. If we mark the derived allele frequencies of variants a and b as q_a and q_b then if $q_a = 1 - q_b$ then $r^2_{\text{max}} = 1$. Since choice in tagging variant can flip the derived allele frequency we only use minor allele frequencies in our inference.

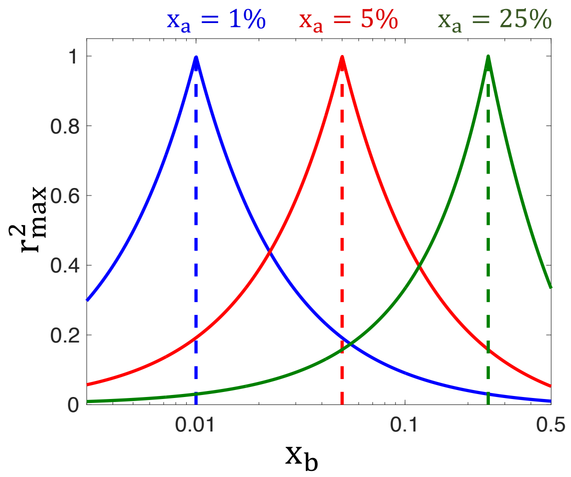

Figure Q: **Maximal correlation. (A)** *The maximal correlation coefficient between two variants a and b as a function of their minor allele frequencies x_a and x_b. Shown are curves for $x_a = 1\%$ (blue), $x_a = 5\%$ (red), and $x_a = 25\%$ (green) with x_b on the x-axis (shown on a log scale).*

7.2 How well does COJO capture the number, frequencies, and effects of hits?

There are multiple heuristic methods for choosing hits and estimating their effect [21], but there isn't much data about how well these methods (and specifically COJO) capture the genetic architecture – i.e., the number, frequencies, and effects of the causal variants the hits tag. In order to test how well COJO captures the genetic architecture, we simulated a height-like genetic architecture using UK biobank GWAS hits for height. We show that COJO hits capture the genetic architecture nearly as well as the causal variants themselves. This happens because in $\sim 90\%$ of cases a hit is in high correlation ($r^2 > 0.8$) with a single causal variant. Furthermore, these simulations also reveal that assortative mating biases the heritability estimates of our method (but not the target size or distribution of selection coefficients estimates).

7.2.1 Simulating hight-like genetic architectures

We use $\sim 330k$ unrelated White British individuals from the UK biobank to run a GWAS on height. We sex-wise rank transformed height to a normal distribution and used PLINK to perform GWAS on the normalized phenotype controlling for 40 PCS, age, sex and age x sex. We then ran COJO on the resulting associations getting 2,158 hits with p-avlue $p < 10^{-6}$ and minor allele frequency $x > 1\%$, 1,134 of these hits passed our filtering for inference inclusion.
We simulated height architecture by taking the full set of 2,158 hits and assuming they're causal and that their effect size is the effect estimated by GWAS + COJO. We then calculated a polygenic score per each of the 330k individuals using these 2,158 hits. We added Gaussian noise to this score such that overall variance would be 1, such that the polygenic score represents the genetic contribution to the simulated phenotype and the noise represents the environmental noise. We then used PLINK to perform GWAS on the simulated phenotype controlling for 40 PCS, age, sex and age x sex and ran COJO on the resulting summary statistics.

7.2.2 Simulation results and the effect of assortative mating

To our initial surprise, the polygenic score produced by our simulation had an $\sim 20\%$ larger variance than expected under an assumption of linkage equilibrium between sites (i.e., than the sum of $2\beta^2 x(1-x)$ over all causal sites). When we ran scores with single variants we saw no excess variance and the excess variance for each chromosome individually was very small. Rather, the excess variance was due to long-range correlation between the genetic scores on different chromosomes. We tried to regress out the 40 PCs of population structure from the score, but the excess variance persisted indicating that it is not related to broad population structure. Instead, we interpret this excess variance as the effect of assortative mating: since the height of parents of the individuals in the UK biobank is correlated then the polygenic scores for height are correlated across chromosomes. This is a well-characterized phenomenon for height and its magnitude has indeed been estimated to be an 18% increase in genetic contribution to the phenotypic variance over the additive contribution and may bias genetic heritability estimates .

As a result of assortative mating, we get more GWAS hits that pass our filtering for inference inclusion for the simulated hight-like trait (1,460 hits) than we do for height (1,134 hits), and their z-scores are systematically larger (see SA&B). In fact, if we look at the causal variants, we see that effect sizes are inflated by a factor of 1.2 throughout (see R).

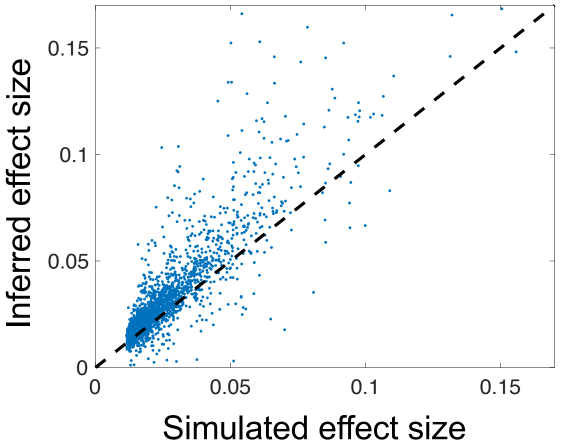

Figure R: **Effect of assortative mating.** *Assortative mating causes a systematic overestimation of effect sizes, since the effect of each SNP also tags some of the effect of assortative mating. Figure data available at: https://doi.org/10.5281/zenodo.17041176.*

7.2.3 Simulation without assortative mating

We used two approaches in order to simulate height-like genetic architectures without assortative mating. In the first approach, we randomly flipped the direction of all alleles within a genome block [6]. In the second approach, we replaced the original SNPs with random frequency matched alleles on the same chromosome. We then repeated the simulation procedure described above.

Both approaches yielded simulated hight-like traits with very similar genetic architectures to height. The number of hits was similar (1,117 for flipped direction, 1,057 for matched alleles, 1,134 for height itself) as were the distributions of minor allele frequencies and effect sizes. In addition, they were virtually identical to those distributions at the causal variants themselves (see Fig. SA&B). This indicates that COJO hits do represent the underlying genetic architecture, i.e. the number,

frequencies and effect sizes of the underlying causal variants.

7.2.4 Tagging of causal variants

We want to understand the extent to which tagging variants are correlated to underlying causal variants. We find that 90% of hits tag a single causal variants at high LD of $r^2 > 0.8$ (94% with assortative mating, 88% with flipped direction , and 89% for matched alleles). Furthermore, a sizable proportion of hits are the causal variant itself (69% with assortative mating, 50% with flipped direction , and 31% for matched alleles).
Though these simulations represent and idealized scenario , they do indicate that COJO is very effective at finding reliable tagging variants for causal variants.

7.2.5 Inference on simulation

We compared the results of our inference for height and for the three simulations. As you can see in Fig. SC-E, the distribution of selection coefficients and the target size are consistent between height itself and the three simulations. However, as seen for other GWAS-based heritability estimates [22], the heritability is upwardly biased by assortative mating by about 20%.
This suggests that while the reliance on GWAS hits doesn't affect our inference results, assortative mating can create an upward bias in heritability estimates. This suggests that our heritability estimates for traits under assortative mating, such as height, are upwardly biased. This might explain why "age at first sexual intercourse" has heritability larger than one in most bootstraps, since age of mates and educational attainment are both known to be under assortative mating and are strongly correlated with "age at first sexual intercourse".
Addressing this bias is beyond the scope of this work.

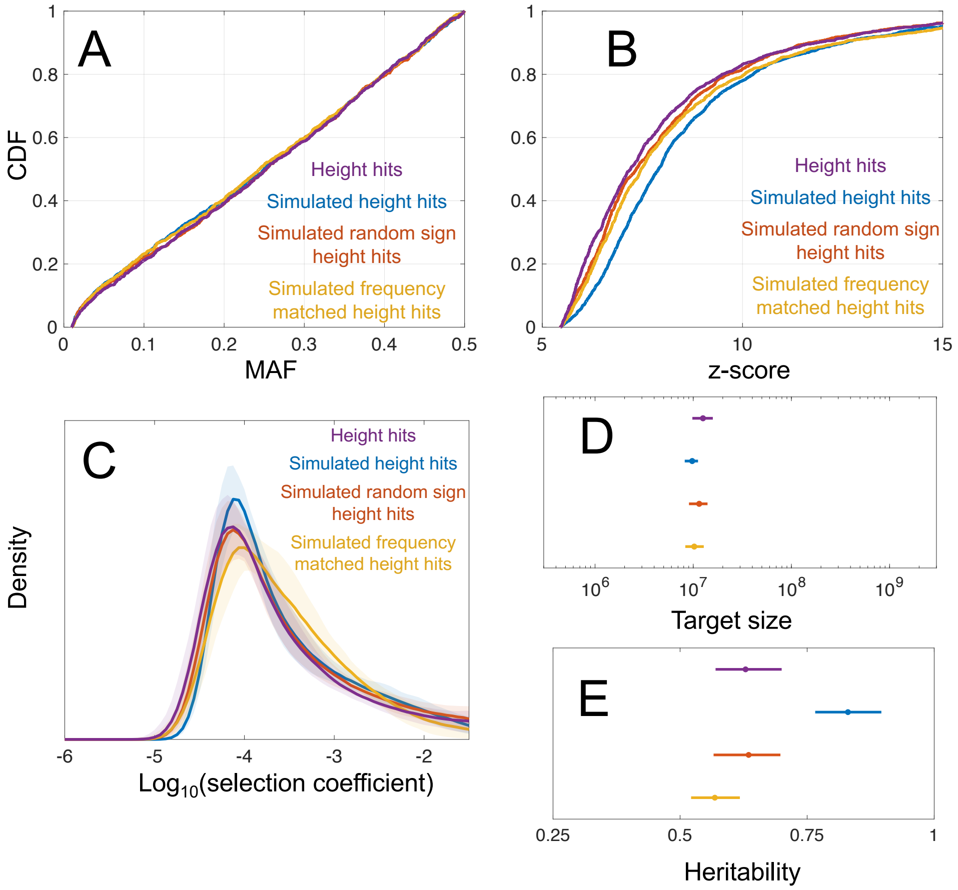

Figure S: **The genetic architecture and inference for simulated architectures.** *We ran simulation of height like genetic architecture using either the GWAS hits for height (in blue), the same hits with the sign of the effect block-wise randomized (in red), or with random frequency-matched alleles replacing the original hits (in orange). When simulating with the original GWAS hits for height, we see an inflation in z-scores (but not MAFs) compared to the hits themselves (A & B). This inflation is eliminated when the sign of the effect is randomized or when we use other SNPs, we therefore interpret it as the effect of assortative mating. While this inflation doesn't effect the distribution of selection coefficients (C) or the estimated target size (D), it upwardly biases our heritability estimates (E). Figure data available at: https://doi.org/10.5281/zenodo.17041176.*

7.3 Are our estimates of the number, frequencies, and effects of hits biased by linked small effect variants?

Each large effect variant is surrounded by many small effect variants whose effect is far from the genome-wide significance threshold. We want to test if small effect variants inflate the effects of large effect variants. In order to test this, we simulate a height-like genetic architecture using the GIANT consortium GWAS hits for height [23], which capture the majority of common variant heritability for height. The logic is that for this architecture there are approximately 10 non-significant variants for each significant variant in the UK biobank. Despite this, GWAS hit effects are not appreciably inflated by background effects.

7.3.1 Simulating hight-like genetic architectures using the GIANT dataset

The GIANT consortium recently reported the results of a meta-analysis GWAS with a sample size of over 5 million individuals from multiple populations [23]. We restricted ourselves to their results for individuals of European origin with a sample size of ~3.9 million individuals. This meta-analysis GWAS resulted in 9,834 COJO hits.
Note, that because this is a meta-analysis that relies on multiple datasets using different genotyping arrays, they report results for only ~ 1.5 million variants (a ten-fold reduction compare to the Neale lab summary statistics for the UK biobank). As a result, the effective study size (reflecting the INFO score) has a much wider distribution than in the UK biobank with more pronounced dependency on MAF. Running our inference on this dataset would require taking these issues into consideration and is beyond the scope of the current work.
We simulated height architecture by taking these 9,834 hits and assuming they're causal and that their effect size is the effect estimated by meta-analysis GWAS + COJO. We then calculated a polygenic score per each of the 330k individuals using these hits. We added Gaussian noise to this score such that overall variance would be 1, such that the polygenic score represents the genetic contribution to the simulated phenotype and the noise represents the environmental noise. We then used PLINK to perform GWAS on the simulated phenotype controlling for 40 PCS, age, sex and age x sex and ran COJO on the resulting summary statistics.

7.3.2 Expected architecture

Because of the differences in study design, we did not want to compare the resulting architecture to what we get for height in the UKBB without the issues arising in a meta-analysis. Instead, we use a simple model of GWAS as the expected distribution against which to check the simulated GWAS results.
The model is simple:

$$z = \sqrt{M \cdot 2x(1-x)} \cdot \beta + \epsilon$$
$$\epsilon \sim N(0,1)$$

with z being the z-score, $M = 336,945$ being the UK biobank study size, x being the MAF, and β the true effect size as defined by our simulation. For simplification, we did not consider the variation in effective study size between variants.

7.3.3 Results

As you can see in T, the simulated GWAS results align very well with the simple GWAS model. Specifically, we do not see an inflation in z-scores despite simulating almost 10 non-significant variants for every GWAS hit and having, on average, almost 6 variants per each genomic block. This suggests that GWAS + COJO handles background effects pretty well, with little inflation of z-scores at GWAS hits.

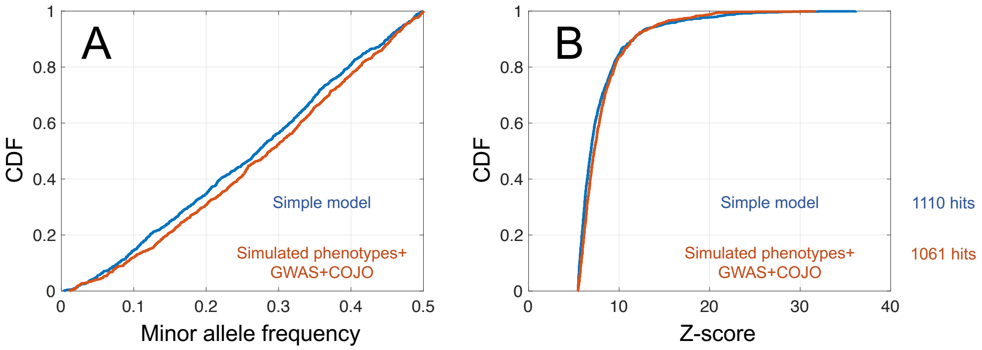

Figure T: **Simulation with 9,834 hits.** *The distribution of minor allele frequencies ((**A**) and z-scores (**B**) of GWAS hits in the UK biobank for phenotypes simulated using polygenic scores with 9,834 hits from GIANT [23] follow the expected distribution. The slight discrepancy in minor allele frequencies is due to the difference in the number of rare variants included in the UK biobank relative to the GIANT dataset. Figure data available at: https://doi.org/10.5281/zenodo.17041176.*

8 Allele ages

In this section, we use the output of RELATE estimation of genome-wide genealogies [4] to show that GWAS hits are younger than matched alleles. While the distribution of allele ages of matched alleles matches our prediction for neutral alleles, the distribution of allele ages for GWAS hits deviates from our predictions. We explain why this deviation is due to a bias in the point estimator for allele ages for alleles under selection. We use simple timescale arguments to suggest a heuristic correction for this bias that resolves this discrepancy.

8.1 RELATE output

At each genomic location, RELATE has estimated the local genealogy among the 1,000 genome samples, spanning a diverse set of global populations. These genealogies are mainly informed by local haplotype structure.

Each variant is then placed on this local genealogy and RELATE reports the branch in the genealogy on which this variant has arisen. For this terminal branch, RELATE reports estimates of the branch's beginning and end, which provide a lower an upper bound on that variant's age. We denote these bounds as T_{low} and T_{up}, see Fig. UA.

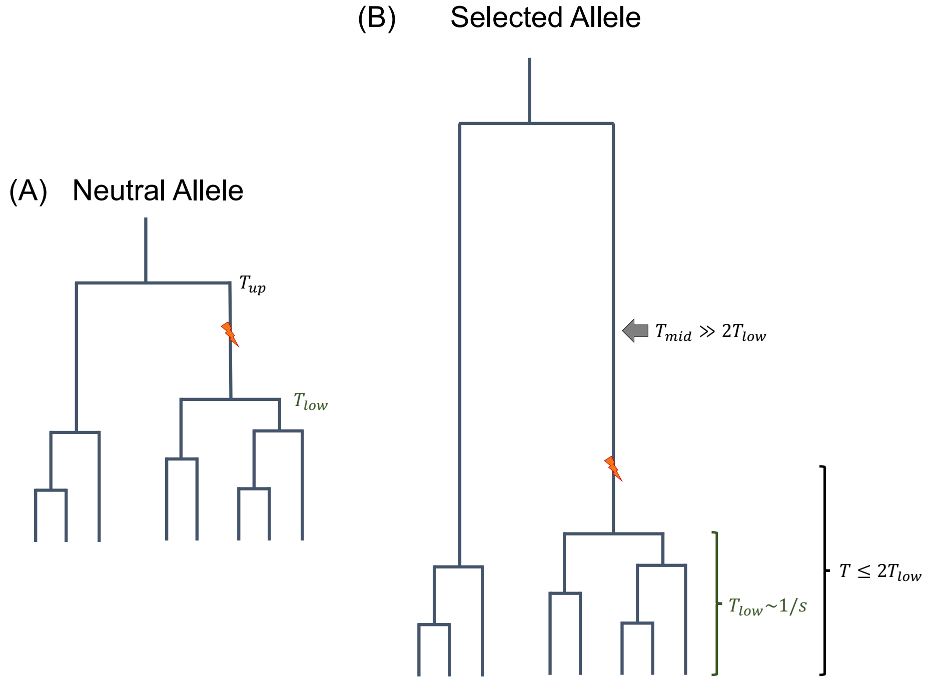

Figure U: **Schematic of mutation origin on terminal branch.** ***(A)*** *By placing a mutation on the genealogy inferred at a locus we can bound the mutation age from above and below by the edges of the branch on which it arose.* ***(B)*** *For an allele under selection, a mutation could only have arisen in the lower part of the branch.*

8.2 GWAS hits are younger than matched controls

RELATE documentation recommends treating the midpoint of the branch on which a variant rose as its age, i.e. $\hat{T}_{mid} = \frac{1}{2}(T_{low} + T_{up})$. We used this estimator to estimate the age of GWAS hits for all 95 traits in our dataset. To see if they are indeed younger than neutral alleles, we matched each hit with a variant of a similar derived allele frequency. We chose the matched SNPs from regions of low background selection (top 10% of B statistic, taken from Murphy et al [24]). We see that indeed GWAS hits are younger than matched alleles (Fig. V).

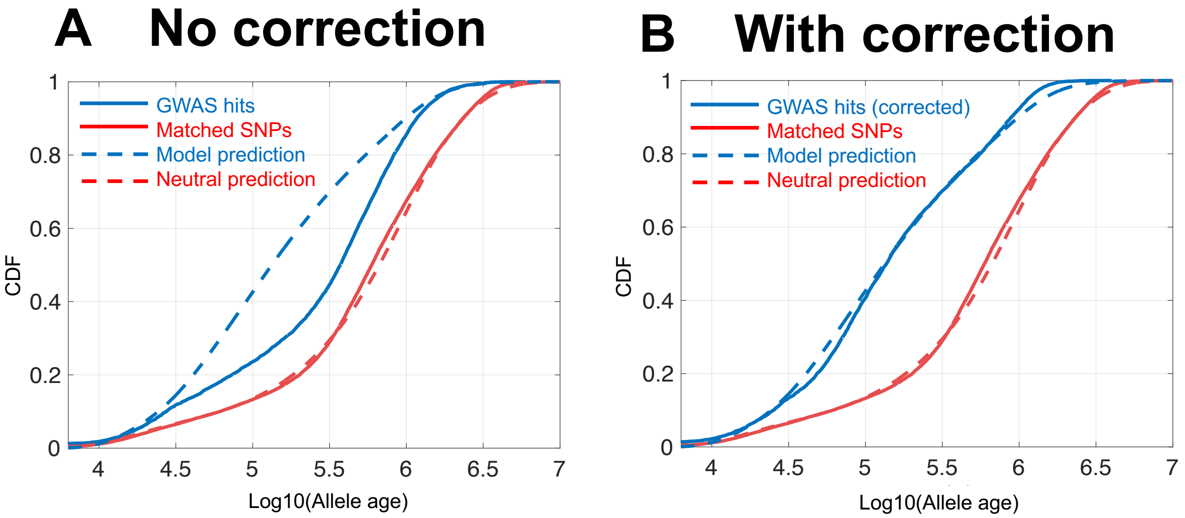

Figure V: **Distribution of allele ages.** ***(A)*** *GWAS hits are younger than matched alleles. While the distribution of allele ages of matched allele ages aligns well with neutral prediction, there is some discrepancy between our prediction for GWAS hits and RELATE based estimates.* ***(B)*** *After applying our heuristic correction, this discrepancy disappears. Figure data available at: https://doi.org/10.5281/zenodo.17041176.*

8.3 Allele age prediction

We next wanted to see if the distribution of allele ages matches our predictions so we ran simulations, under our demographic model, for different selection coefficients. For each variant, we recorded its frequency and age. Then, for each selection coefficient and frequency bin we could estimate the distribution of allele ages $P(T|x, s)$.

As a first step, we show that our predictions for neutral alleles match what we see for matched SNPs. For neutral predictions, we estimate the distribution of allele ages for variants with the same MAFs as our GWAS hits. We use $s = 10^{-6}$ as a proxy for neutrality. For GWAS hit i, we have a distribution of age conditional on its frequency $P(T|x_i, s = 10^{-6})$. The overall distribution is just an average over the distributions for each SNP

$$p_{neut}(T) = \frac{1}{n}\sum_i P(T|x_i, s = 10^{-6})$$

with n being the total number of hits. Our prediction for neutral alleles closely matches the distribution of allele ages produced by the midbranch point estimator T_{mid}, see Fig. V.

Next, we want to compare the predictions of our inferred model to the observed distribution. We again obtain a distribution per SNP, conditioning on it being a hit:

$$p(T|hit, C_i, x) = \frac{P(T|x, s)P(hit|C_i, x, s)P(x|s)f(s)}{\int_s P(T|x, s)P(hit|C_i, x, s)P(x|s)f(s)}$$

This prediction does not fully align with what we observe, see Fig. V.

8.4 Bias in allele age estimation

RELATE gives us an estimate of the two edges of the terminal branch on which an allele arose in the genetic genealogy. For a neutral allele, RELATE documentation suggests the midpoint of this branch as an estimator of allele age. However, this makes little sense for common alleles under selection.

Many GWAS hits originate in long branches, with the branch midpoint more than twice the branch starting point T_{low}, see Fig. UB. Such branches exist due to a combination of the out-of-Africa bottleneck, population structure within Africa before the bottleneck and the limited resolution the 1000 genomes dataset gives for the genetic genealogy before the out-of-Africa exodus.

Unlike a neutral allele, a selected allele could not have arisen at any point on the branch. After arising by mutation, a selected variant either goes extinct after a few generations or rises in frequency on a timescale of $1/s$ generations. Regardless of the mode and direction of selection, selected variants have sojourn times of the order of $1/s$. The age of such a variant is then smaller (in order of magnitude) then $T_{low} + 1/s$. Assuming the variant is presently common and present in multiple samples in a dataset, the time until all copies coalesce, T_{low}, is also of the order of $1/s$. The conclusion is that the age of such a variant is smaller then $2 \cdot T_{low}$ (at least in order of

magnitude). That is, when a selected alleles maps to a long branch, it must have arisen at the lower part of the branch, see Fig. UB.

8.5 Heuristic point estimate of allele age

We found that a simple heuristic correction solves the bias. We sought a point estimate of allele ages, $\hat{T}$, that satisfies $\hat{T} - T_{low} \leq T_{low}$. The simple estimator we use is just to take $\hat{T} - T_{low} = \frac{1}{2} \cdot \min(T_{high} - T_{low}, T_{low})$. This estimator resolves the discrepancy, see Fig. V and Fig. 5 in the main text.

We did however test a whole family of estimators of the form $\hat{T}_\lambda = T_{low} + \frac{1}{2} \cdot \min(T_{high} - T_{low}, \lambda T_{low})$., such that $\hat{T}_1$ is the estimator we use in the main text and we have the limits $\hat{T}_0 = T_{low}$ and $\hat{T}_\infty = T_{mid}$. In Fig. W, you can see that our estimates are not sensitive to the exact choice of λ, with all $\lambda \sim 1$ giving similar results. If λ is too small, the estimator becomes insensitive to T_{high} and allele ages are downwardly biased. If λ is too big, long branches create a bias in allele ages.

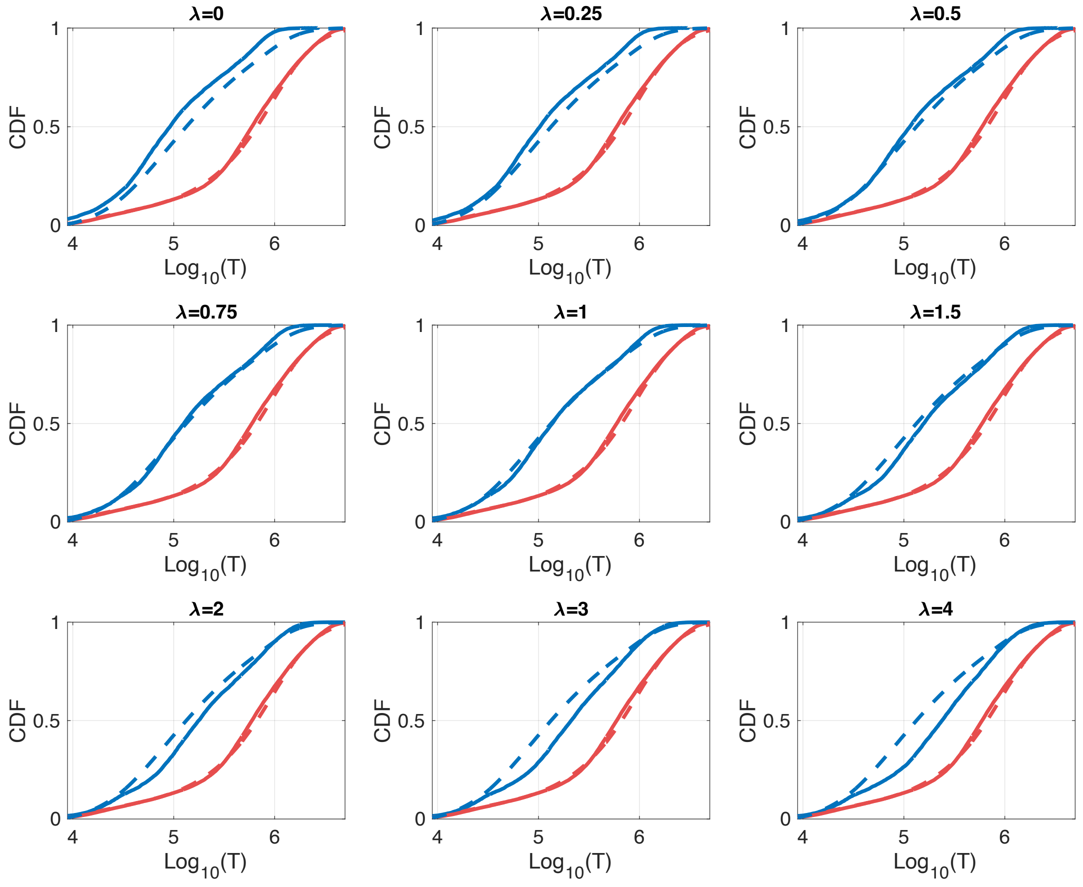

Figure W: **Heuristic estimators of allele ages.** *We tried different variations of our heuristic estimator and we see that for a wide range of* λ *values the distributions of allele ages closely matches our prediction. Figure data available at: https://doi.org/10.5281/zenodo.17041176.*

8.6 Sensitivity to estimates of s

To test how sensitive our predictions are to the inferred distribution of selection coefficients, we tested the accuracy of our predictions when $f(s)$ is shifted up or down by $10^{0.5}$ (Fig. XA). As you can see in Fig. XB, these shifts in selection coefficient result in shifts in the distribution of allele ages such that the predicted distributions no longer match the observed distribution.

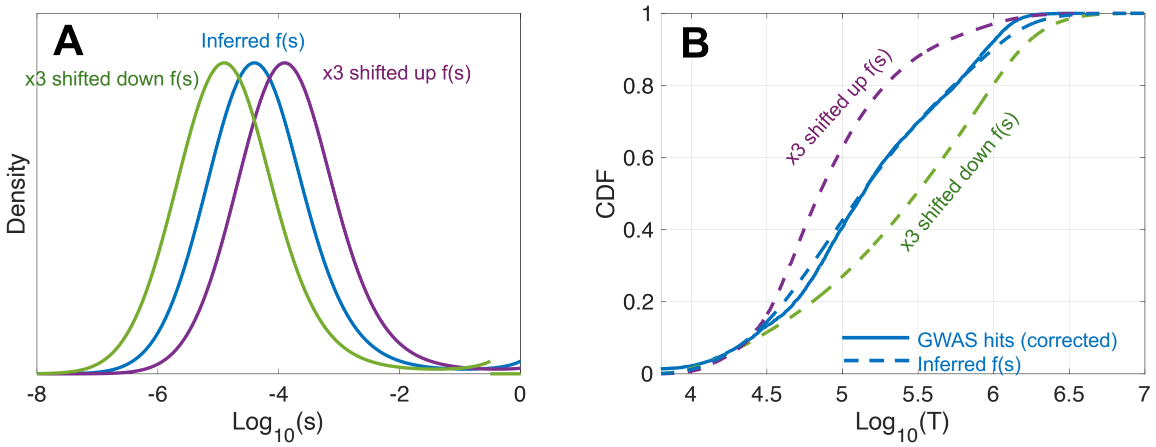

Figure X: **Prediction of allele ages with shifted distributions of selection coefficients.** *(A) We take the inferred distribution of selection coefficients under the SDS model and shift it up (in purple) or down (in green) by* $10^{0.5}$*. (B) The resulting predicted allele ages are shifted and no longer match the observed distribution of allele ages for GWAS hits. Figure data available at: https://doi.org/10.5281/zenodo.17041176.*

9 Parameter values in main text figures

9.1 Parameter values for Figure 1

C&D: The selection coefficient are $10^{-4.5}$ for neutral, 10^{-3} for moderate selection, and 10^{-2} for strong selection. $c = 10^4$.

9.2 Parameter values for Figure 2

For all panels, trait 1 has $h^2 = 75\%$, $L = 30$Mbp and a normal distribution of $\log_{10}(s)$ with mean $\log_{10}(s) = -4$ and standard deviation of 0.5.

Trait 2 has the following parameters:
A&D: $h^2 = 0.75$ and $L = 30$Mbp and a normal distribution of $\log_{10}(s)$ with mean $\log_{10}(s) = -3$ and standard deviation of 0.5.
B&E: $h^2 = 0.25$ and $L = 30$Mbp and a normal distribution of $\log_{10}(s)$ with mean $\log_{10}(s) = -4$ and standard deviation of 0.5.
A&D: $h^2 = 0.25$ and $L = 10$Mbp and a normal distribution of $\log_{10}(s)$ with mean $\log_{10}(s) = -4$ and standard deviation of 0.5.

9.3 Parameter values for Figure 6

We use the distribution of selection coefficients, $f(s)$, inferred using the SSD model (see Fig. 3). For study size, we use that of the UK biobank British population (360,000 individuals). The low h^2/L is $3 \cdot 10^{-8}$ and the high h^2/L is $3 \cdot 10^{-7}$.

10 Similarities in genetic architectures after scaling

In this section, we provide figures analogous to Fig. 7 of the main text, but only for effect sizes. We look at all traits with median study size above $3.3 \cdot 10^5$. We focus on one trait at each row and plot in blue the CDF of effect sizes for that trait. In grey, we plot the CDF of effect sizes for all other traits with h^2/L bigger than that of the focal trait.

We then scale the effect sizes and plot the CDF of the scaled effect sizes for the focal trait (in blue) and all the other traits with h^2/L bigger than that of the focal trait together (in grey). We join the signal for all traits together to reduce noise, since after scaling and thresholding we are left with only a few hits for some traits. Data for the resulting figures available at: https://doi.org/10.5281/zenodo.17041176.

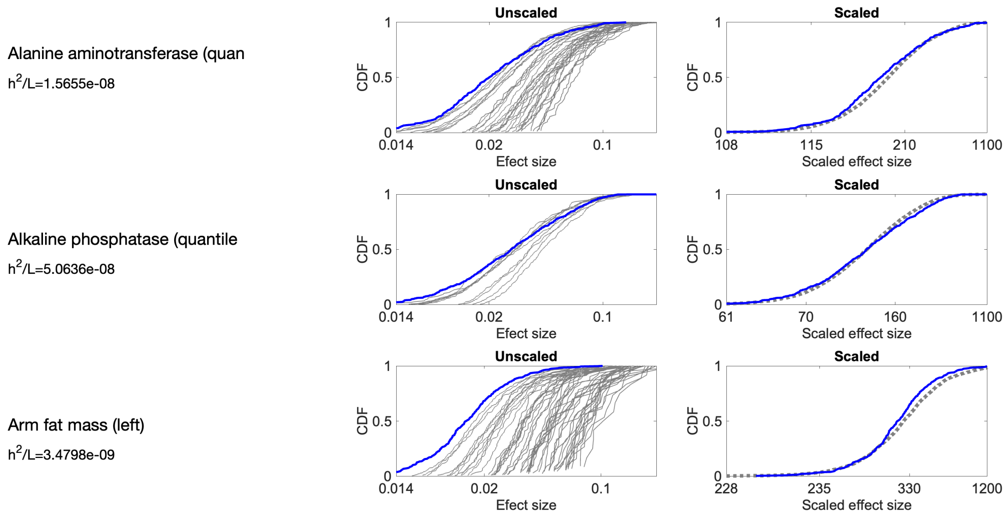

Arm fat percentage (left)
h^2/L=2.6394e-09

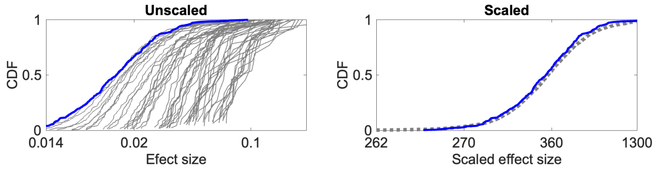

Arm fat-free mass (right)
h^2/L=8.5405e-09

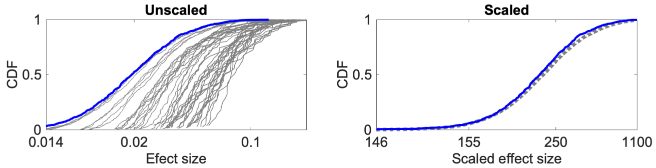

Arm predicted mass (right)
h^2/L=9.1514e-09

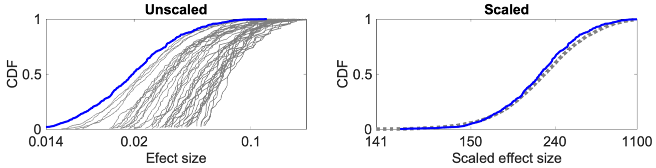

Basal metabolic rate
h^2/L=8.5384e-09

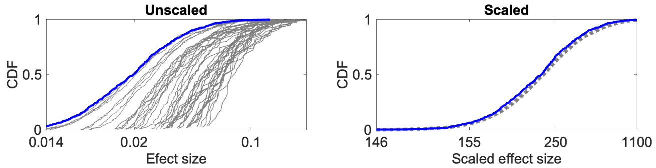

Basophill percentage
h^2/L=2.6151e-08

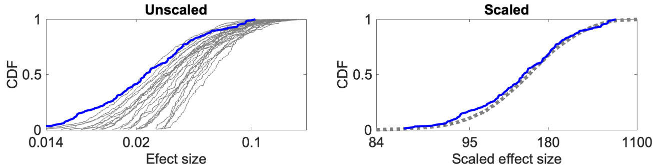

Body fat percentage
h^2/L=2.7644e-09

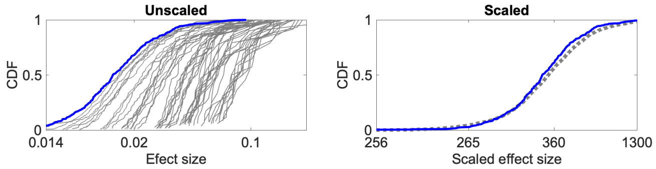

Body mass index (BMI)
h^2/L=3.1997e-09

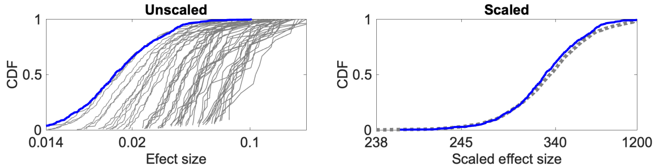

Body mass index (BMI)
h^2/L=3.1722e-09

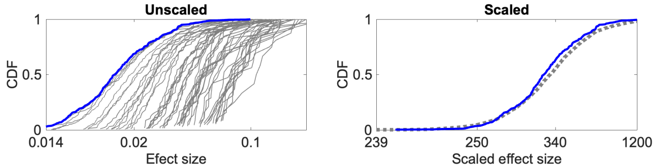

Creatinine (quantile)
h^2/L=1.4952e-08

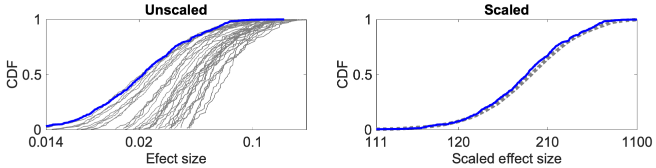

Diastolic blood pressure, auto

h^2/L=4.5677e-09

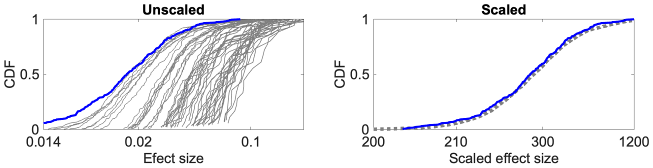

Eosinophill percentage

h^2/L=2.9342e-08

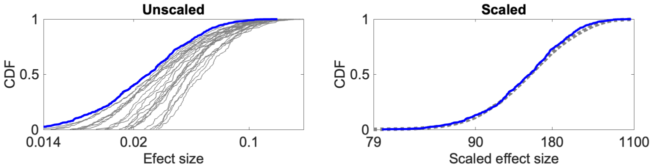

Gamma glutamyltransferase (qua

h^2/L=4.5154e-08

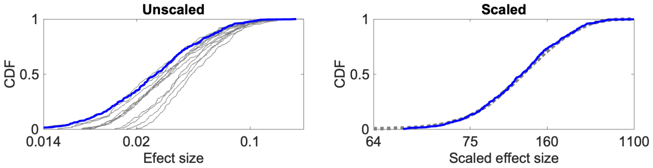

Glycated haemoglobin (quantile

h^2/L=3.5043e-08

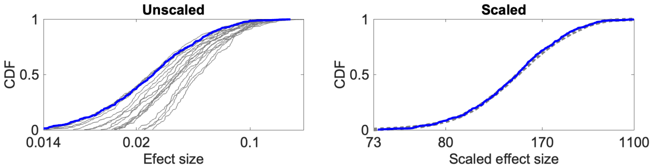

Haematocrit percentage

h^2/L=1.6471e-08

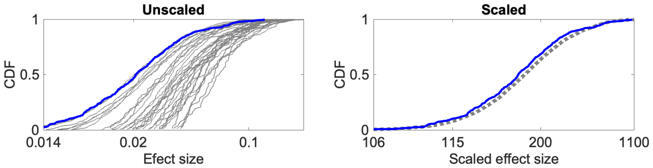

Haemoglobin concentration

h^2/L=1.6471e-08

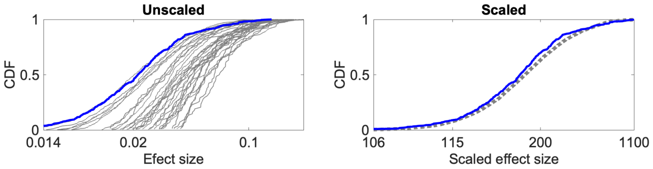

Hand grip strength (right)

h^2/L=1.5682e-09

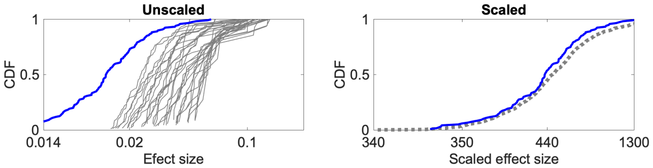

High light scatter reticulocyt

h^2/L=3.2652e-08

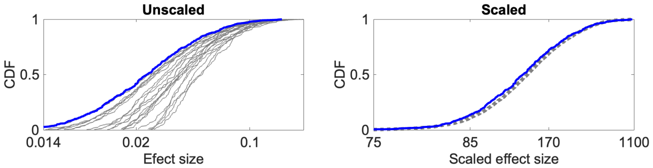

High light scatter reticulocyt

h^2/L=3.6636e-08

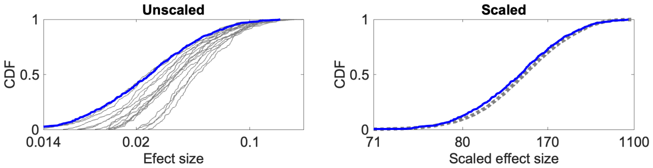

Hip circumference

h^2/L=5.0635e-09

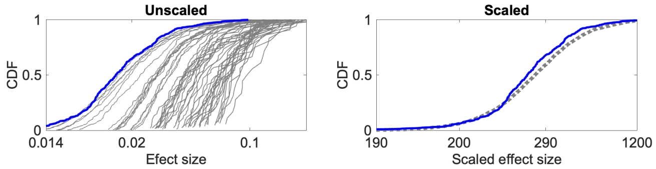

IGF-1 (quantile)

h^2/L=1.9806e-08

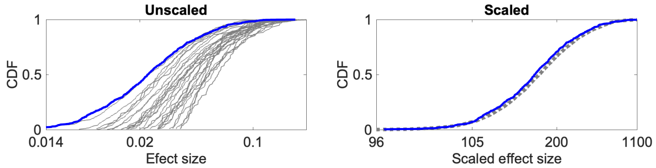

Immature reticulocyte fraction

h^2/L=3.7489e-08

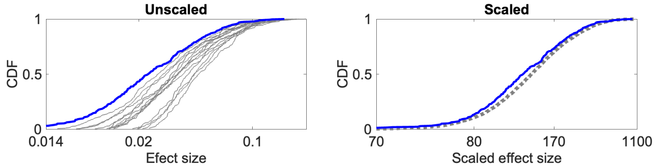

Impedance of arm (left)

h^2/L=6.0449e-09

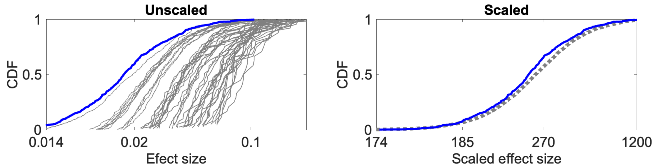

Impedance of leg (left)

h^2/L=5.2649e-09

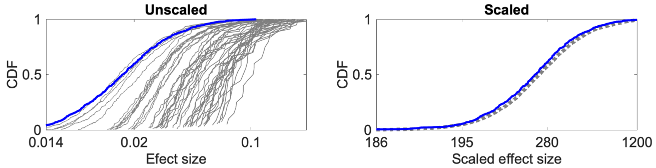

Impedance of whole body

h^2/L=5.773e-09

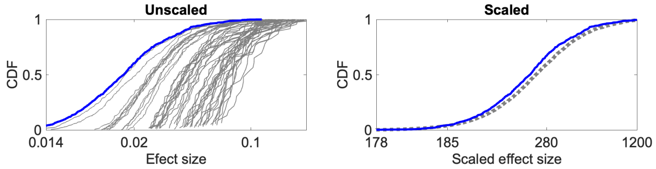

Leg fat mass (left)

h^2/L=3.4787e-09

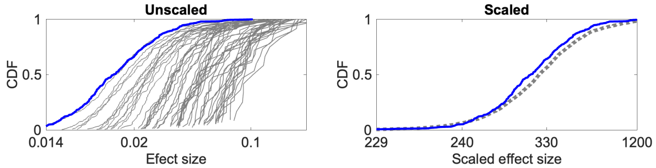

Leg fat percentage (left)

h^2/L=2.7003e-09

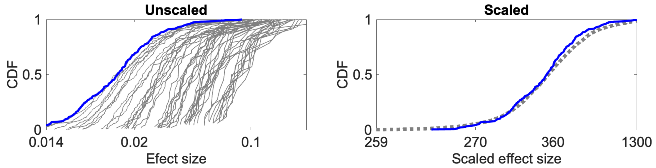

Leg fat-free mass (right)

h^2/L=8.9413e-09

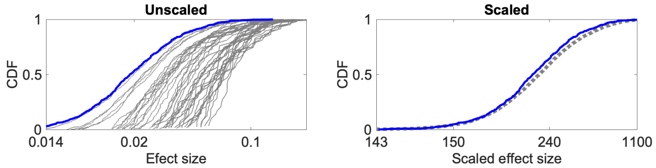

Leg predicted mass (right)

h^2/L=8.9413e-09

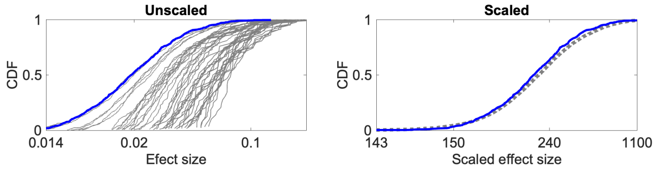

Lymphocyte count

h^2/L=1.9838e-08

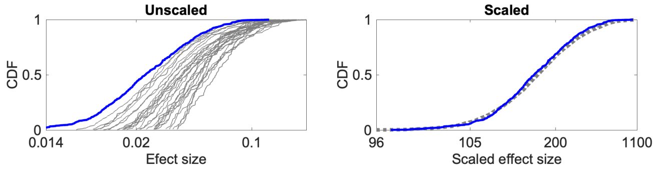

Lymphocyte percentage

h^2/L=1.768e-08

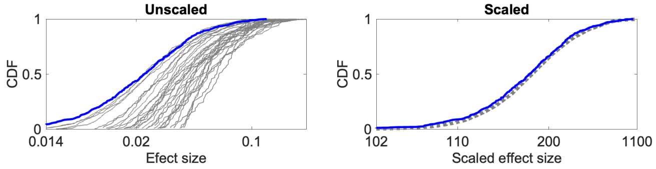

Mean corpuscular haemoglobin c

h^2/L=5.2087e-08

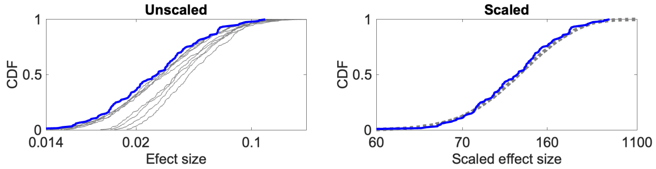

Mean corpuscular volume

h^2/L=8.0674e-08

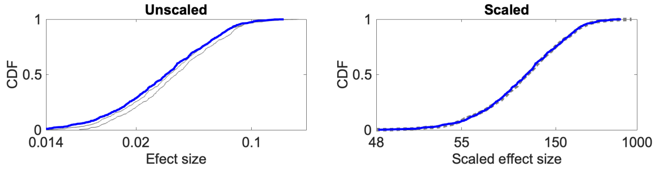

Mean platelet (thrombocyte) vo

h^2/L=8.6444e-08

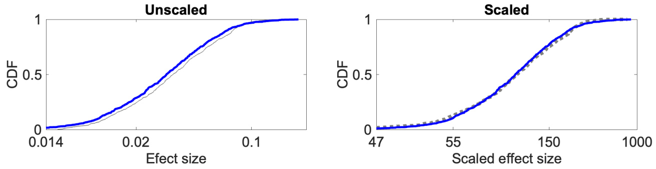

Mean reticulocyte volume

h^2/L=7.48e-08

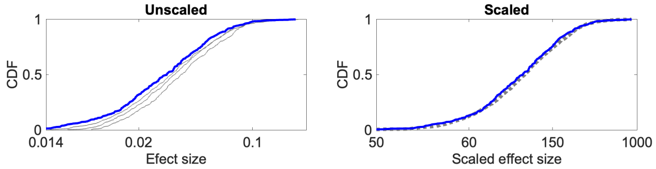

Mean sphered cell volume

h^2/L=5.9416e-08

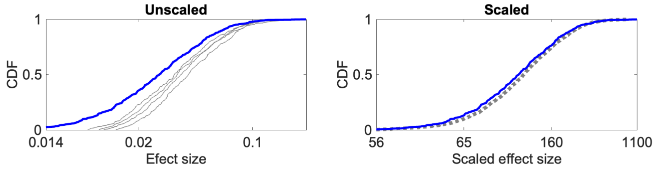

Monocyte count

h^2/L=3.5277e-08

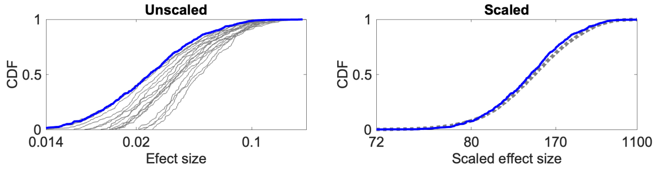

Monocyte percentage

h^2/L=5.7212e-08

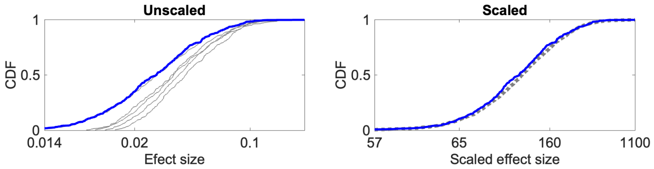

Neutrophill count

h^2/L=1.8514e-08

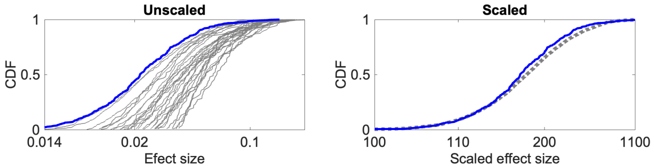

Neutrophill percentage

h^2/L=1.4706e-08

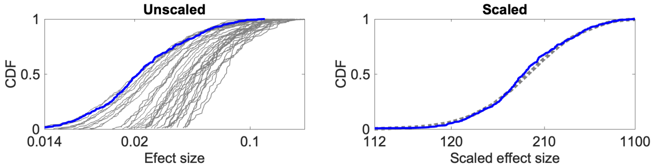

Platelet count

h^2/L=4.5366e-08

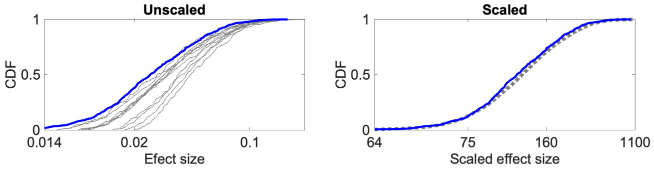

Platelet crit

h^2/L=3.0671e-08

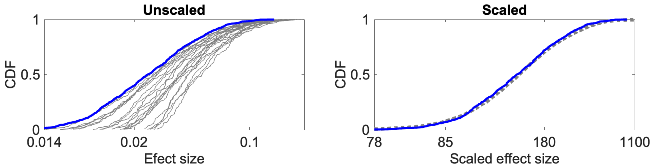

Platelet distribution width

h^2/L=5.7113e-08

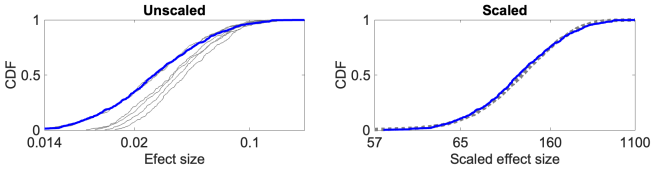

Pulse rate, automated reading

h^2/L=1.4444e-08

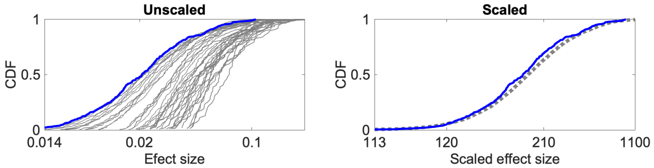

Red blood cell (erythrocyte) c

h^2/L=2.4362e-08

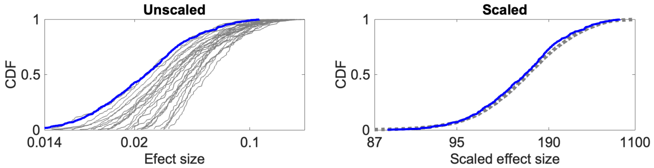

Red blood cell (erythrocyte) d

h^2/L=5.5813e-08

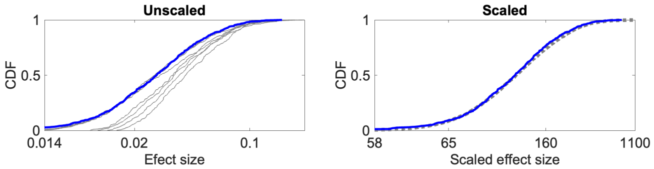

Reticulocyte count

h^2/L=3.7489e-08

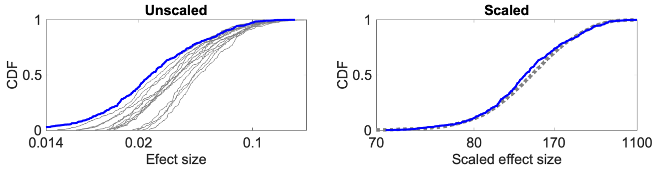

Reticulocyte percentage

h^2/L=4.2063e-08

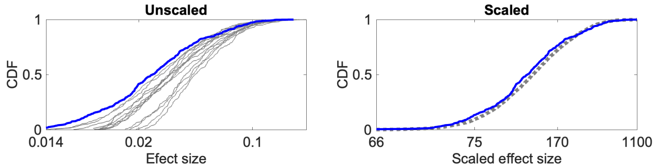

Sitting height

h^2/L=1.8408e-08

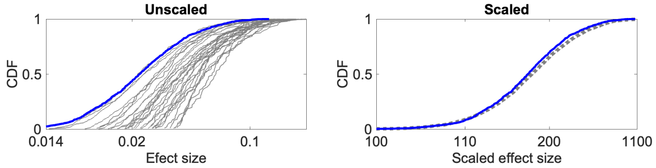

Standing height

h^2/L=2.7203e-08

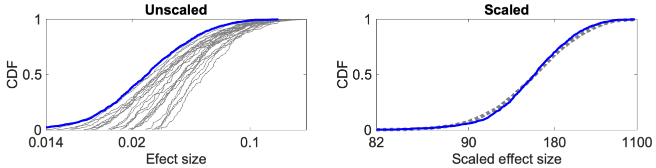

Systolic blood pressure, autom

h^2/L=4.1658e-09

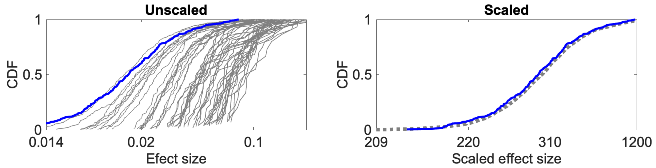

Triglycerides (quantile)

h^2/L=3.2722e-08

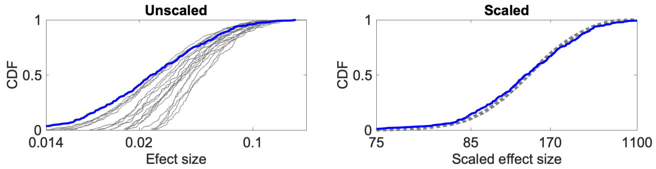

Trunk fat mass

h^2/L=3.9962e-09

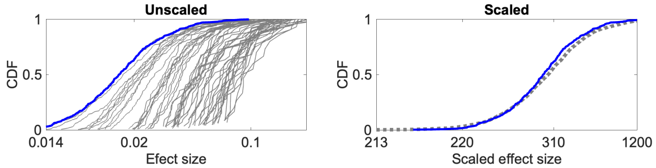

Trunk fat percentage

h^2/L=2.9622e-09

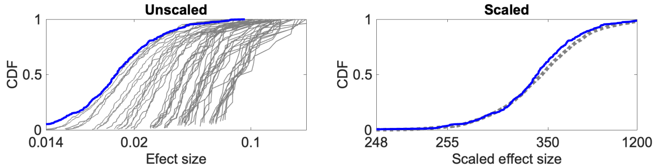

Trunk fat-free mass

h^2/L=1.1527e-08

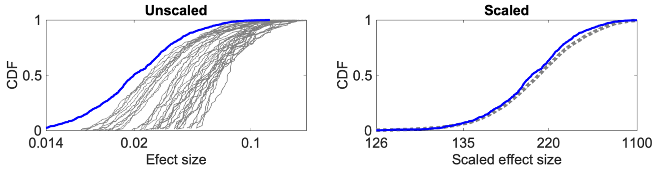

Trunk predicted mass

h^2/L=1.1266e-08

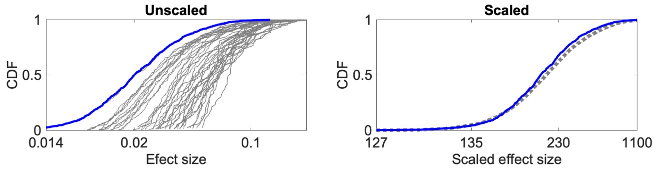

Urea (quantile)

h^2/L=2.6593e-08

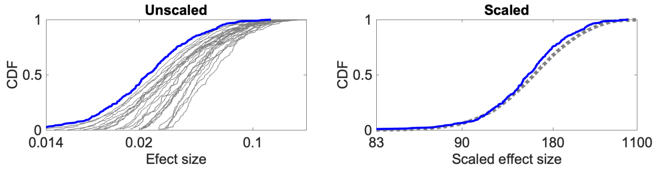

Waist circumference

h^2/L=2.8471e-09

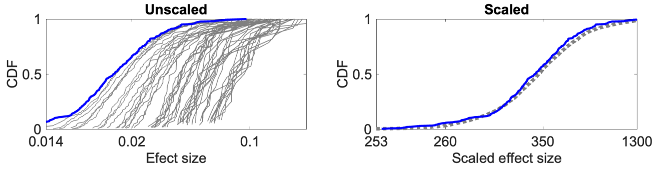

Weight

h^2/L=5.6877e-09

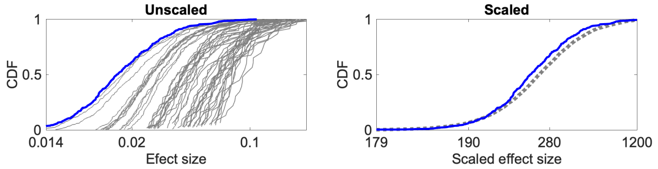

Weight

h^2/L=5.387e-09

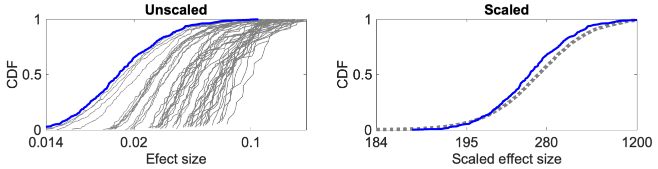

White blood cell (leukocyte) c

h^2/L=1.6471e-08

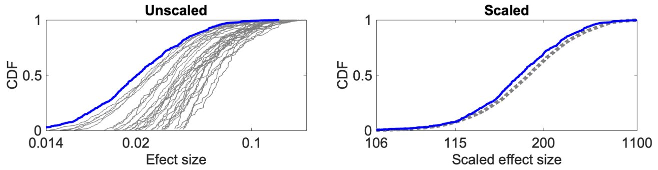

Whole body fat mass

h^2/L=4.1888e-09

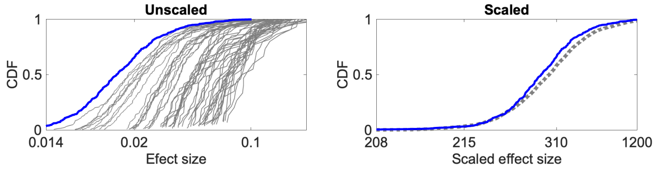

Whole body fat-free mass

h^2/L=1.0505e-08

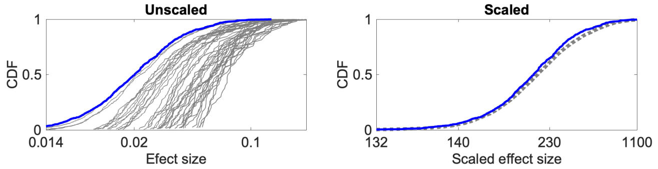

Whole body water mass

h^2/L=1.0749e-08

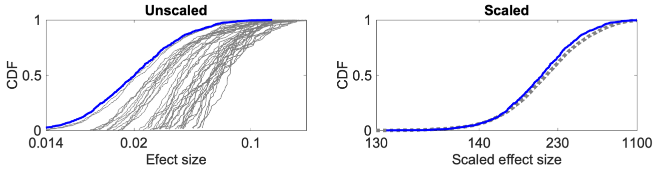

11 Supplementary tables

11.1 Table A: Outlier SNPs

CHR	BP	Traits	Gene	rsid	Type of Mutation
1	248039451	Immature reticulocyte fraction	TRIM58	rs3811444	missense
2	27730940	Triglycerides (quantile)	GCKR	rs1260326	missense
6	26104632	Haemoglobin concentration	SLC17A2	rs386698213	noncoding
7	46753491	IGF-1 (quantile)	IGFBP3	rs700750	noncoding
10	71094504	Haematocrit percentage<br>Haemoglobin concentration	HK1	rs17476364	intron
14	94844947	Albumin (quantile)	SERPINA1	rs28929474	missense
16	53802494	Arm fat mass (left)<br>Arm fat percentage (left)<br>Body fat percentage<br>Body mass index (BMI)<br>Hip circumference<br>Impedance of leg (left)<br>Impedance of whole body<br>Leg fat mass (left)<br>Leg fat percentage (left)<br>Trunk fat mass<br>Waist cirumference<br>Weight<br>Whole body fat mass	FTO	rs1421085	intron
17	7080316	Alkaline phosphatase (quantile)	ASGR1	rs55714927	synonymous
20	4157072	Immature reticulocyte fraction	SMOX	rs6084653	intron
22	37462936	Haemoglobin concentration	TMPRSS6	rs855791	missense
22	44324727	Alanine aminotransferase (quantile)	PNPLA3	rs738409	missense
22	46364161	3mm strong meridian (left)<br>3mm weak meridian (left)<br>6mm strong meridian (left)<br>6mm weak meridian (left)	WNT7B	rs9330813	intron

For an **expanded version of this table (Table S2)**, which includes our explanation of the mechanism by which each SNP directly affects the trait(s) for which it an outlier, **see the attached xlsx file**.

11.2 Table B: Model and inference assumptions

Assumption	Support	Effect of violation
The frequencies and effect sizes of underlying causal variants well-captured by GWAS hits	See supplement Section 7 for comprehensive analysis	Unknown bias in the co-distribution of frequency and effect sizes
GWAS hits represent a censored, but otherwise unbiased, subset of the genetic architecture	Other then being common and with large effect, nothing suggests GWAS hits are unrepresentative	Unknown bias in the co-distribution of frequency and effect sizes
High pleiotropy	Variants routinely being implicated in multiple GWAS; PWAS; Large target size imply overlap b/w traits	Overestimation of heritability, target size and width of f(s)
Similar level of pleiotropy among all hits for a given trait	May not be globally true, but model fit suggests it it is a reasonable assumption	Underestimation of heritability, target size and width of f(s)
Trait mean close to trait optimum	Theory suggests any deviation from optimum is small and short-lived; Little observed difference between trait-increasing and trait-decreasing variants	Difference in genetic architecture between trait-increasing and trait-decreasing variants
Trait is continuous	Some traits are continuous; However, many biomedically relevant traits are not	Model and inference would have to be tweaked to accommodate diseases and other categorical phenotypes

References

[1] Simons YB, Bullaughey K, Hudson RR, Sella G. A population genetic interpretation of GWAS findings for human quantitative traits. PLOS Biology. 2018;16(3):e2002985.

[2] Simons YB, Turchin MC, Pritchard JK, Sella G. The deleterious mutation load is insensitive to recent population history. Nature Genetics. 2014;46(3):220-4. Available from: https://doi.org/10.1038/ng.2896.

[3] Yang J, Ferreira T, Morris AP, Medland SE, Madden PAF, Heath AC, et al. Conditional and joint multiple-SNP analysis of GWAS summary statistics identifies additional variants influencing complex traits. Nature Genetics. 2012;44(4):369-75. Available from: https://doi.org/10.1038/ng.2213.

[4] Speidel L, Forest M, Shi S, Myers SR. A method for genome-wide genealogy estimation for thousands of samples. Nature Genetics. 2019;51(9):1321-9. Available from: https://doi.org/10.1038/s41588-019-0484-x.

[5] Earl DJ, Deem MW. Parallel tempering: Theory, applications, and new perspectives. Phys Chem Chem Phys. 2005;7:3910-6. Available from: http://dx.doi.org/10.1039/B509983H.

[6] Berisa T, Pickrell JK. Approximately independent linkage disequilibrium blocks in human populations. Bioinformatics. 2015 09;32(2):283-5. Available from: https://doi.org/10.1093/bioinformatics/btv546.

[7] Min J, Chiu DT, Wang Y. Variation in the heritability of body mass index based on diverse twin studies: a systematic review. Obesity Reviews. 2013;14(11):871-82. Available from: https://onlinelibrary.wiley.com/doi/abs/10.1111/obr.12065.

[8] Hunter DJ, De Lange M, Snieder H, Mac Gregor AJ, Swaminathan R, Thakker RV, et al. Genetic contribution to renal function and electrolyte balance: a twin study. Clinical Science. 2002 08;103(3):259-65. Available from: https://doi.org/10.1042/cs1030259.

[9] Ingebrigtsen TS, Thomsen SF, van der Sluis S, Miller M, Christensen K, Sigsgaard T, et al. Genetic Influences on Pulmonary Function: A Large Sample Twin Study. Lung. 2011 Aug;189(4):323-30. Available from: https://doi.org/10.1007/s00408-011-9306-3.

[10] Silventoinen K, Sammalisto S, Perola M, Boomsma DI, Cornes BK, Davis C, et al. Heritability of Adult Body Height: A Comparative Study of Twin Cohorts in Eight Countries. Twin Research. 2003;6(5):399–408.

[11] Marroni F, Grazio D, Pattaro C, Devoto M, Pramstaller P. Estimates of Genetic and Environmental Contribution to 43 Quantitative Traits Support Sharing of a Homogeneous Environment in an Isolated Population from South Tyrol, Italy. Human Heredity. 2007 10;65(3):175-82. Available from: https://doi.org/10.1159/000109734.

[12] Tarnoki AD, Szalontai L, Fagnani C, Tarnoki DL, Lucatelli P, Maurovich-Horvat P, et al. Genetic and environmental factors on heart rate, mean arterial pressure and carotid intima–media thickness: A longitudinal twin study. Cardiology Journal. 2021;28(3):431 438. Available from: https://journals.viamedica.pl/cardiology_journal/article/view/CJ.a2019.0089.

[13] Yeh LK, Chiu CJ, Fong CF, Wang IJ, Chen WL, Hsiao CK, et al. The Genetic Effect on Refractive Error and Anterior Corneal Aberration: Twin Eye Study. Journal of Refractive Surgery. 2007;23(3):257-65. Available from: https://journals.healio.com/doi/abs/10.3928/1081-597X-20070301-08.

[14] Kettunen J, Tukiainen T, Sarin AP, Ortega-Alonso A, Tikkanen E, Lyytikäinen LP, et al. Genome-wide association study identifies multiple loci influencing human serum metabolite levels. Nature Genetics. 2012 Mar;44(3):269-76. Available from: https://doi.org/10.1038/ng.1073.

[15] Menni C, Mangino M, Zhang F, Clement G, Snieder H, Padmanabhan S, et al. Heritability analyses show visit-to-visit reflects different pathological phenotypes in younger and older adults: evidence from UK twins. Journal of Hypertension. 2013;31(12). Available from: https://journals.lww.com/jhypertension/fulltext/2013/12000/heritability_analyses_show_visit_to_visit_blood.7.aspx.

[16] Pathan N, Deng WQ, Di Scipio M, Khan M, Mao S, Morton RW, et al. A method to estimate the contribution of rare coding variants to complex trait heritability. Nature Communications. 2024 Feb;15(1):1245. Available from: https://doi.org/10.1038/s41467-024-45407-8.

[17] Purcell S, Neale B, Todd-Brown K, Thomas L, Ferreira MAR, Bender D, et al. PLINK: A Tool Set for Whole-Genome Association and Population-Based Linkage Analyses [doi: 10.1086/519795]. The American Journal of Human Genetics. 2007 2022/09/04;81(3):559-75. Available from: https://doi.org/10.1086/519795.

[18] Kanai M, Elzur R, Zhou W, analysis Initiative GBM, Daly MJ, Finucane HK. Meta-analysis fine-mapping is often miscalibrated at single-variant resolution. medRxiv. 2022. Available from: https://www.medrxiv.org/content/early/2022/08/22/2022.03.16.22272457.

[19] Wang G, Sarkar A, Carbonetto P, Stephens M. A Simple New Approach to Variable Selection in Regression, with Application to Genetic Fine Mapping. Journal of the Royal Statistical Society Series B: Statistical Methodology. 2020 07;82(5):1273-300. Available from: https://doi.org/10.1111/rssb.12388.

[20] VanLiere JM, Rosenberg NA. Mathematical properties of the r2 measure of linkage disequilibrium. Theoretical Population Biology. 2008;74(1):130-7. Available from: https://www.sciencedirect.com/science/article/pii/S0040580908000609.

[21] Uffelmann E, Huang QQ, Munung NS, De Vries J, Okada Y, Martin AR, et al. Genome-wide association studies. Nature Reviews Methods Primers. 2021;1(1):59.

[22] Border R, O'Rourke S, de Candia T, Goddard ME, Visscher PM, Yengo L, et al. Assortative mating biases marker-based heritability estimators. Nature communications. 2022;13(1):660.

[23] Yengo L, Vedantam S, Marouli E, Sidorenko J, Bartell E, Sakaue S, et al. A saturated map of common genetic variants associated with human height. Nature. 2022;610(7933):704-12.

[24] Murphy D, Elyashiv E, Amster G, Sella G. Broad-scale variation in human genetic diversity levels is predicted by purifying selection on coding and non-coding elements. bioRxiv. 2021. Available from: https://www.biorxiv.org/content/early/2021/09/28/2021.07.02.450762.
